# GPR182 is a lipoprotein receptor for dietary fat absorption

**DOI:** 10.1172/JCI200857

**Published:** 2026-03-24

**Authors:** Zhiwei Sun, Robert J. Torphy, Emily N. Miller, Anza Darehshouri, Isaac Vigil, Taichi Terai, Eleanor Eck, Yi Sun, Yujie Guo, Dustin P. Fykstra, Elliott J. Yee, Junyi Hu, Ross M. Kedl, Erika L. Lasda, Jay R. Hesselberth, Julie A. Siegenthaler, Paul S. MacLean, Kimberley D. Bruce, Gwendalyn J. Randolph, Richard D. Schulick, Yuwen Zhu

**Affiliations:** 1 Department of Surgery,; 2 Department of Cell and Developmental Biology,; 3 Department of Immunology and Microbiology,; 4 Department of Biochemistry and Molecular Genetics,; 5 Department of Pediatrics, Section of Developmental Biology, and; 6 Division of Endocrinology, Metabolism, and Diabetes, Department of Medicine, the University of Colorado Anschutz Medical Campus (UC AMC), Aurora, Colorado, USA.; 7 Department of Pathology and Immunology, Washington University School of Medicine, St. Louis, Missouri, USA.

**Keywords:** Metabolism, Vascular biology, Lipoproteins, Lymph, Obesity

## Abstract

The lymphatic system plays a central role in lipid absorption by transporting triglyceride-rich particles called chylomicrons (CMs) from the small intestine to the systemic circulation. However, the molecular mechanism by which CMs get into the intestinal lymphatics is unknown. Here, we demonstrated that GPR182, an atypical chemokine receptor in lymphatic endothelial cells, mediates dietary fat absorption. GPR182-KO mice exhibited a selective increase in circulating high-density lipoproteins and are resistant to diet-induced obesity. GPR182 ablation in mice led to poor lipid absorption and thereby a delay in growth during development. GPR182 broadly interacted with and transported lipoproteins. Transmission electron microscopy analysis revealed that, mechanistically, loss of GPR182 prevented CMs from entering the lacteal lumen of the small intestine. Consistent with this, GPR182 blockade with mAbs protected mice from diet-induced obesity and treated existing obesity. Together, our study identifies GPR182 as a lipoprotein receptor that mediates dietary fat absorption and supports GPR182 blockade as a feasible approach to treating obesity and related disorders.

## Abstract 2

This study shows that dietary fat absorption in the small intestine depends on a receptor called GPR182.

## Introduction

Lipid transport, facilitated by lipoproteins and their corresponding receptors, plays a pivotal role in distributing essential nutrients and endogenously synthesized lipids to various tissues (1). Dysregulation of these processes, or lipid disorders, are major risk factors for metabolic diseases, which include, but are not limited to, obesity, atherosclerosis, fatty liver disease, type 2 diabetes, and neurodegenerative disorders (2, 3). Unlike nutrients such as amino acids and simple sugars that directly enter the bloodstream, lipid absorption involves the lymphatic system (4, 5). In the small intestine, dietary fatty acids are repackaged into particles called chylomicrons (CMs) before entering the lymphatic structure known as lacteals (6). Despite its critical importance as a necessary step in fat absorption, the process through which CMs enter into lacteals is poorly understood (7). The prevailing view assumes that CMs reach the lacteal lumen through open cell-to-cell junctions between lymphatic endothelial cells (LECs), known as “button junctions,” which allow the passage of large cargoes like CMs (8–10). The conversion of intestinal lacteals from button-like to zipper-like junction leads to defects in fat absorption (10, 11). However, a receptor-mediated transcytosis mechanism for CM transport has not yet been excluded (12–14).

GPR182 is a newly characterized atypical chemokine receptor (ACKR) of the GPCR superfamily (15–17). GPR182 is primarily expressed by microvascular endothelial cells, sinusoidal endothelial cells, and LECs (15, 18, 19), and GPR182 interacts with chemokines broadly via the glycosaminoglycan-binding (GAG-binding) motif (16, 17, 20). In this study, we found that GPR182 broadly interacted with lipoproteins and that ablation of GPR182 in mice led to resistance to high-fat diet–induced (HFD-induced) obesity. Our further study supports the notion that GPR182 serves as a receptor to mediate dietary fat absorption in the small intestine.

## Results

### Gpr182^–/–^ mice are protected from diet-induced obesity.

Adult young *Gpr182^–/–^* mice on a C57BL/6J background were healthy as they grew and bred normally on a regular diet, consistent with previous reports (16, 18). However, we observed that *Gpr182^–/–^* mice weighed consistently less than their WT littermates as they aged, with the difference between male mice being more prominent (Supplemental Figure 1A; supplemental material available online with this article; https://doi.org/10.1172/JCI200857DS1). Male *Gpr182^–/–^* mice over 5-month-old became visibly slimmer in body shape (Supplemental Figure 1B). Examination of body composition via MRI confirmed that male *Gpr182^–/–^* mice had a selective reduction in fat mass with increased age (Supplemental Figure 1C).

**Figure 1:**
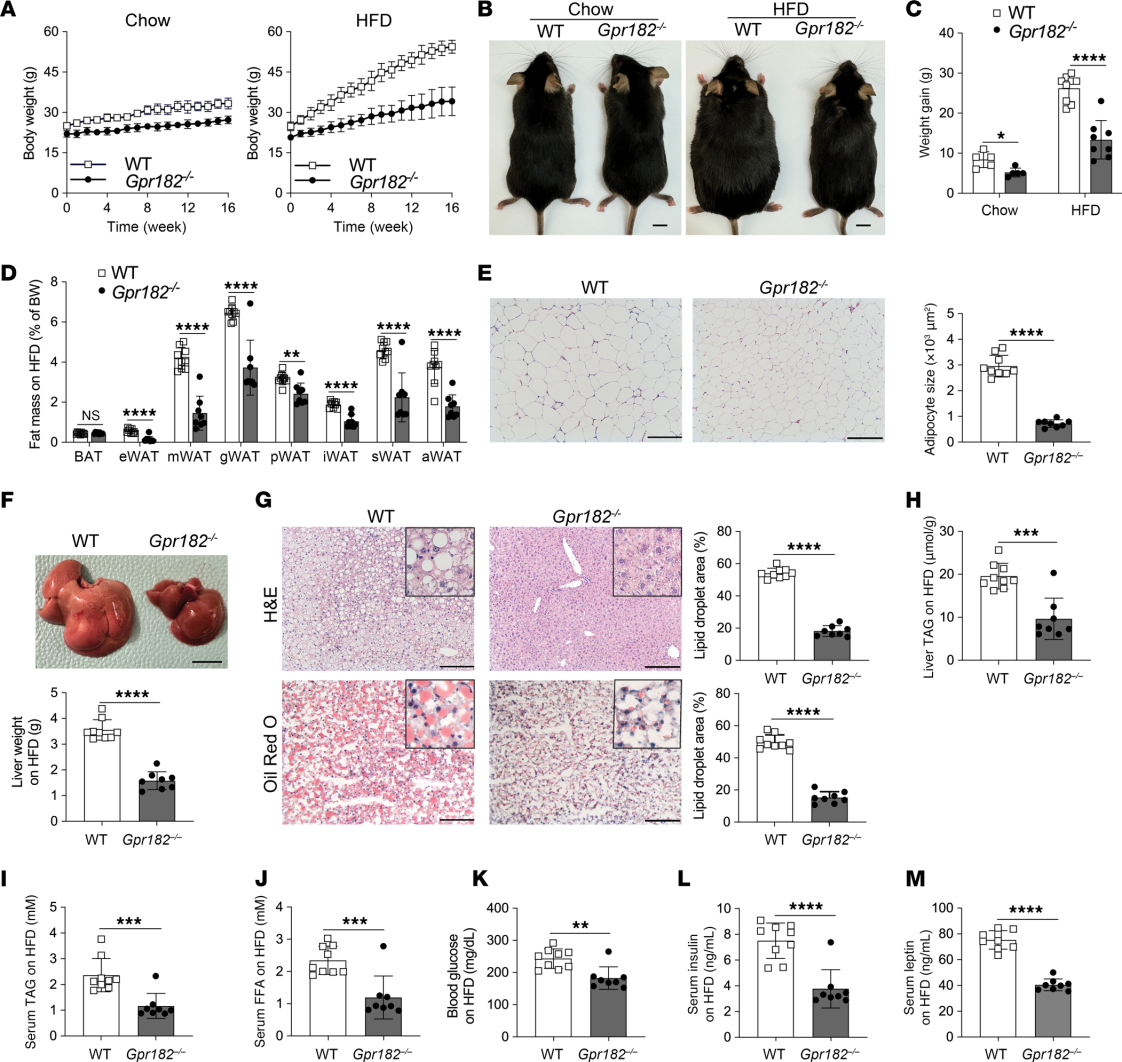
*Gpr182^–/–^* mice are resistant to diet-induced obesity. Seven-week-old male WT and *Gpr182*^–/–^ mice were fed a chow diet or a HFD for 16 weeks. (**A**) BW was followed weekly. (**B**) Representative images of mice following 16 weeks of HFD or chow feeding (scale bars: 1 cm) and (**C**) BW gain. (**D**) Fat percentages for WT and Gpr182^–/–^ mice fed a HFD were calculated. eWAT, epicardial WAT; iWAT, inguinal WAT; sWAT, subcutaneous WAT; aWAT, axillary WAT. (**E**) H&E staining of gWAT from *Gpr182^–/–^* and control mice on a HFD (scale bars: 200 μm) and adipocyte sizes. (**F**) Representative images of livers from *Gpr182*^–/–^ and WT mice on a HFD (scale bar: 1 cm) and liver weights. (**G**) H&E and Oil Red O staining of liver tissues from *Gpr182^–/–^* and WT control mice on a HFD. Scale bars: 200 μm. Insets, original magnification: ×400. TAG levels in the liver (**H**) and serum levels of TAG (**I**), FFA (**J**), glucose (**K**), insulin (**L**), and leptin (**M**) in Gpr182^–/–^ and control WT mice on a HFD were quantified. Chow diet: *n* = 5; HFD: *n* = 8–9, pooled from 2 independent experiments. Data represent the mean ± SEM. An unpaired, 2-tailed Student’s *t* test was used to compare the means of 2 groups (**C**–**M**). *P < 0.05, **P < 0.01, ***P < 0.001, and ****P < 0.0001.

The lymphatic system is known to mediate fat absorption, and GPR182 is primarily expressed in LECs (15, 16). The selectively reduced fat mass in adult *Gpr182^–/–^* mice compelled us to carefully examine the role of GPR182 in dietary fat absorption. We monitored weight gain for young adult WT and *Gpr182^–/–^* mice over a 16-week period of a standard chow diet or a HFD. At 7 weeks of age, *Gpr182^–/–^* male mice on a chow diet weighed slightly less than age-matched WT controls (Figure 1A). Over 16 weeks of a chow diet, *Gpr182^–/–^* mice gained weight at a slower pace than did the WT controls (Figure 1, A–C). The most striking observation was the substantial resistance of *Gpr182^–/–^* mice to HFD-induced obesity (Figure 1A and 1B). Following 16 weeks of HFD feeding, male *Gpr182^–/–^* mice gained approximately 13 g in BW, whereas WT control mice doubled their BW (weight gain of >26 g/mouse, Figure 1C). Consequently, the fat mass in *Gpr182^–/–^* mice was far less than that of WT controls (Figure 1D). White adipose tissues (WAT) samples from *Gpr182^–/–^* mice were much smaller and weighed proportionally less than those from WT mice (Figure 1D). There was no difference in brown adipose tissue (BAT) between *Gpr182^–/–^* and WT mice (Figure 1D). Histological analysis confirmed adipocyte hypertrophy in WT mice upon 16 weeks of HFD feeding, and adipocytes from HFD-fed *Gpr182^–/–^* mice were markedly smaller (Figure 1E). Livers from *Gpr182^–/–^* mice under HFD appeared normal and weighed less than half of those from WT counterparts (Figure 1F). H&E and Oil Red O staining confirmed that *Gpr182^–/–^* mouse livers contained notably smaller and fewer lipid droplets than did those of WT mice (Figure 1G). Consistently, triacylglycerol (TAG) levels in *Gpr182^–/–^* livers were only approximately 50% of those in WT mouse livers (Figure 1H).

The levels of TAG and free fatty acid (FFA) in serum from fasting *Gpr182^–/–^* mice after 16 weeks of HFD feeding were only approximately 50% of those in serum from WT control mice (Figure 1, I and J). Further, *Gpr182^–/–^* mice exhibited significantly lower concentrations of circulating glucose, insulin, and leptin levels (Figure 1, K–M). Together, our results strongly support the idea that GPR182 ablation protects mice from diet-induced obesity and its related disorders.

### GPR182 ablation prevents lipid absorption in the small intestine.

**Figure 2:**
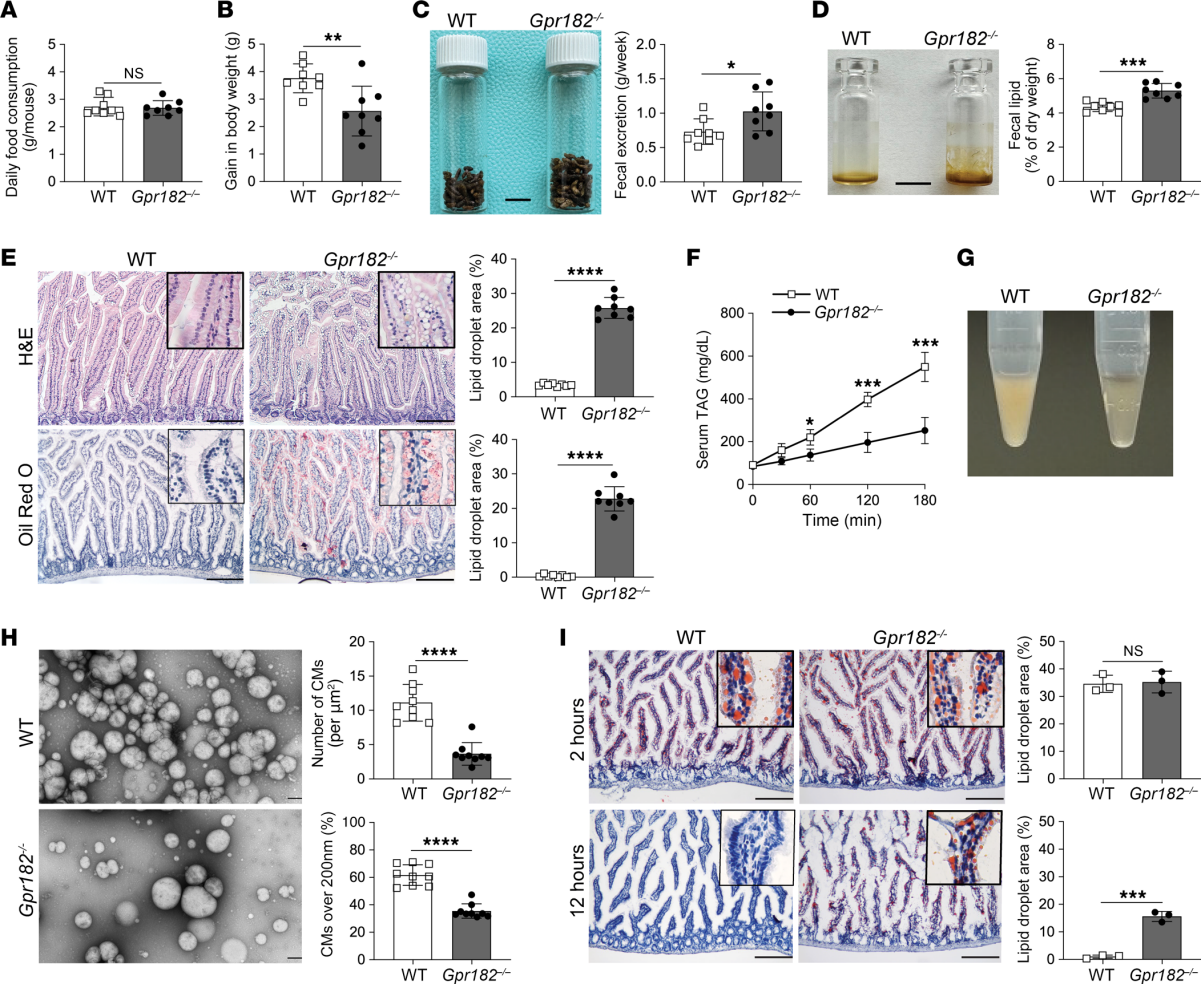
GPR182 ablation limits lipid absorption in the small intestine. (**A**–**D**) Age-matched adult male WT and *Gpr182^–/–^* mice were fed a HFD for 2 weeks. Data shown are representative of 3 independent experiments. Daily food consumption (**A**), BW gain (**B**), feces production (**C)**, and fecal lipids (**D**) were measured (*n* = 8). Scale bars: 1 cm. (**E**) Jejuna from WT and *Gpr182^–/–^* mice on a 2-week HFD were stained with H&E and Oil Red O (*n* = 8). Scale bars: 200 μm. Insets, original magnification: ×400. (**F**–**I**) Fasted female WT and *Gpr182^–/–^* mice were orally gavaged with olive oil. (**F**) TAG levels in serum from *Gpr182^–/–^* and WT mice were quantified over a 3-hour period following olive oil gavage (*n* = 5). (**G**) Representative images of serum from WT and *Gpr182^–/–^* mice 2 hours after oil gavage. (**H**) TEM analysis of CMs in mesenteric lymph fluid from WT and *Gpr182^–/–^* mice 2 hours after oil gavage. The number and size of CMs were quantified. Each symbol represents 1 field (*n* = 3). Scale bars: 200 nm. (**I**) Oil Red O staining of small intestines 2 hours and 12 hours after oil oral gavage was performed and quantified (*n* = 3). Scale bars: 200 μm. Insets, original magnification: ×400. Data represent the mean ± SEM. Two-way ANOVA with Bonferroni’s correction for multiple comparisons was used to compare groups over time by repeated measures (**F**). An unpaired, 2-tailed Student’s *t* test was used to compare the means of 2 groups (**A**–**E**, **H**, and **I**). *P < 0.05, **P < 0.01, ***P < 0.001, and ****P < 0.0001.

To better understand how *Gpr182^–/–^* mice were resistant to HFD-induced obesity, we performed energy balance assessment for mice during 2 weeks of HFD feeding. *Gpr182^–/–^* mice on a HFD had the same daily food intake as their WT counterparts (Figure 2A), whereas they gained less weight in a 2-week span of HFD feeding (Figure 2B). Energy expenditure (Supplemental Figure 2A) and O_2_ consumption (Supplemental Figure 2B) during both the light and dark circadian phases were comparable between *Gpr182^–/–^* and WT mice. A slight increase in CO_2_ production was observed in *Gpr182^–/–^* mice only during the dark phase (Supplemental Figure 2C). Thus, differences in energy consumption probably do not account for the markedly reduced weight gain in *Gpr182^–/–^* mice (Figure 2B). We found that *Gpr182^–/–^* mice produced significantly more feces (Figure 2C), even though their food consumption was the same. Feces produced by *Gpr182^–/–^* mice contained over 20% more residual lipids than those from the WT control group (Figure 2D), suggesting that loss of GPR182 reduced intestinal lipid uptake. As a result, *Gpr182^–/–^* mice on 2 weeks of HFD showed lipid droplet accumulation in the small intestine, which was not found in the control WT mice (Figure 2E). This implies that *Gpr182^–/–^* mice had poor lipid absorption, which led to the increase in lipid excretion via feces.

To further evaluate dietary lipid absorption, we performed oral gavage of olive oil in fasting mice, coadministering tyloxapol to inhibit lipoprotein catabolism. As expected, serum TAG levels in WT mice spiked sharply after oil gavage. In contrast, *Gpr182^–/–^* mice maintained lower levels of serum TAG throughout the assessment (Figure 2F). Consistently, serum from WT mice 2 hours after oil gavage appeared creamy white, whereas *Gpr182^–/–^* serum remained clear (Figure 2G). We collected lymph fluid from mesenteric lymphatics 2 hours after oil gavage and performed transmission electron microscopy (TEM). Lymph liquid from *Gpr182^–/–^* mice contained considerably fewer CM particles than that from WT mice, and CM particles from *Gpr182^–/–^* mice were generally smaller (Figure 2H). Oil Red O staining showed that both WT and *Gpr182^–/–^* mice had lipid-filled small intestines 2 hours after gavage; however, by 12 hours, only *Gpr182^–/–^* mice still exhibited clear lipid droplet accumulation in the small intestine (Figure 2I). These findings indicate a marked impairment in lipid absorption in the absence of GPR182. In further support of that finding, when mice were orally administrated a fluorescent fatty acid analog (BODIPY FL C16), we found significantly weaker fluorescence signal in the mesenteric lymph ducts of *Gpr182^–/–^* mice (Supplemental Figure 2D). On the other hand, the draining function of intestinal lymphatics was comparable between WT and *Gpr182^–/–^* mice when we assessed it by injection of FITC-dextran into the Peyer’s patch (Supplemental Figure 2E). Thus, our studies show that *Gpr182^–/–^* mice had a selective defect in dietary lipid absorption.

### Gpr182^–/–^ mice have normal CM synthesis and lacteal structure.

The small intestine is the main organ for mediating dietary lipid absorption, whereby digested lipid is reassembled into CM particles and then enter the intestinal lymphatics (21). During the process, TAG is synthesized by diacylglycerol acyltransferase 2 (DGAT2) in the smooth ER and then transferred into the rough ER by microsomal triglyceride transfer protein (MTP) (22, 23). In the ER lumen, synthesized TAG and phospholipids interact with ApoB-48 to form CM particles (21, 24). Our analysis of a public single-cell RNA-seq dataset (25) found that GPR182 is exclusively transcribed by LECs in the small intestine (Supplemental Figure 3A and S3B). Immunofluorescence staining of small intestine further confirmed specific expression of GPR182 protein in Lyve1^+^ lacteals (Supplemental Figure 3C) (15, 19). The expression levels of ApoB-48, MTP, and DGAT2 in the small intestine were comparable between WT and *Gpr182^–/–^* mice (Supplemental Figure 3, D and E), suggesting that GPR182 ablation did not affect CM assembly. This was consistent with normal Oil Red O staining in the small intestine of *Gpr182^–/–^* mice at an early stage of oil oral gavage (Figure 2I). *Gpr182^–/–^* mice on a regular chew diet had normal small intestine and colon lengths (Supplemental Figure 3F). There was no alteration in villus or lacteal lengths in adult *Gpr182^–/–^* mice (Supplemental Figure 3G). *Gpr182^–/–^* newborn pups showed normal villi/lacteals and revealed lacteal LEC proliferation comparable to that in WT pups (Supplemental Figure 3, H and I). VE-cadherin staining in intestinal lacteals revealed a similar button-like/zipper-like pattern between adult *Gpr182^–/–^* and control WT mice (Supplemental Figure 3J). All these observations suggest that the poor lipid absorption in *Gpr182^–/–^* mice was not caused by its effect on CM synthesis or lacteal structure.

### GPR182 interacts with lipoproteins broadly.

**Figure 3:**
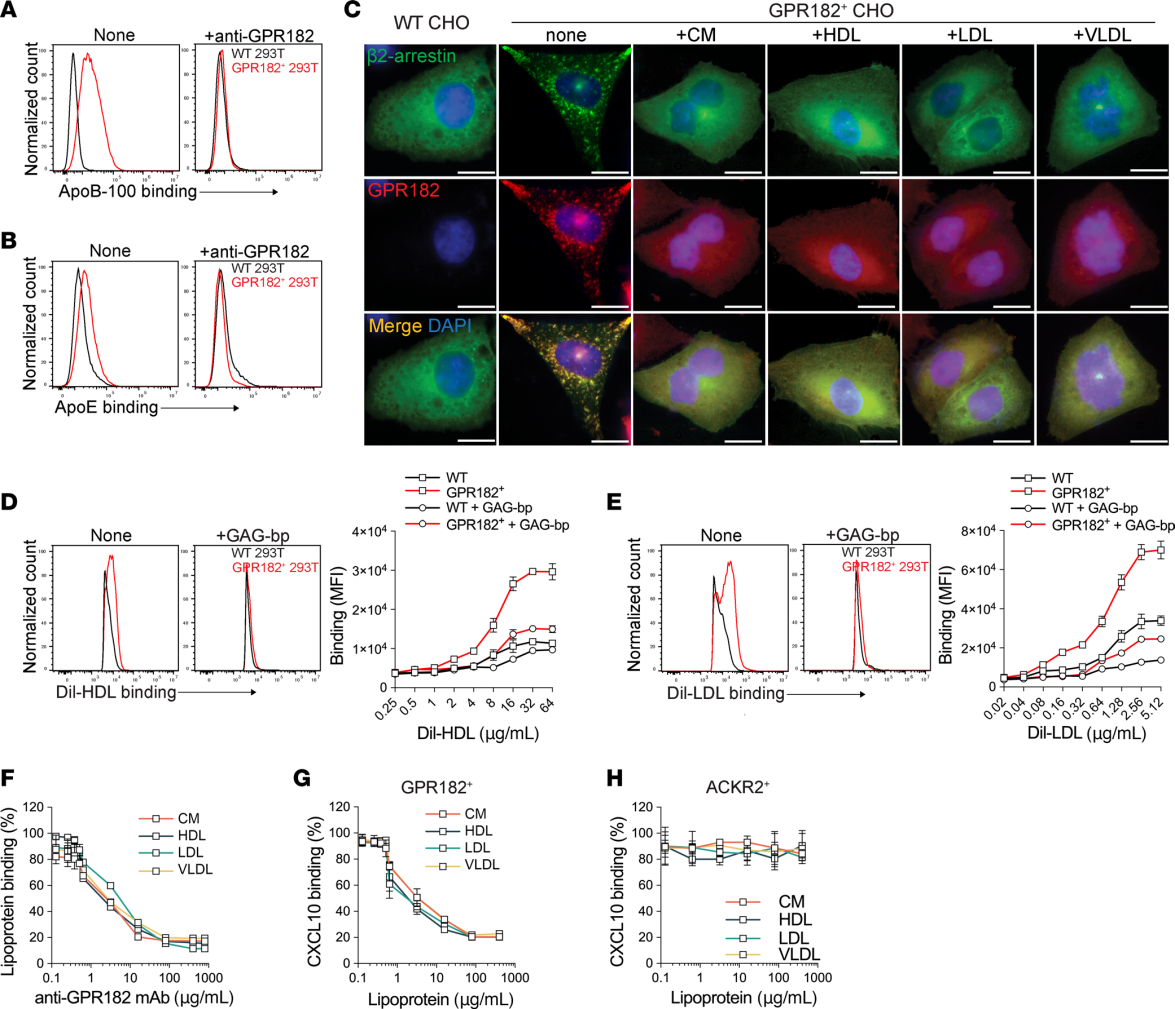
GPR182 interacts broadly with lipoproteins. (**A** and **B**) GPR182^+^ HEK293T and control cells were examined by flow cytometry for protein ApoB (**A**) and ApoE (**B**) binding. GPR182 mAb was added to assess its blocking capacity. (**C**) GPR182^+^ CHO and control CHO cells were transfected to express β-arrestin-2–EGFP. GPR182^+^ CHO cells were incubated with CM, HDL, LDL, or VLDL at 37°C for 1 minute before immunofluorescence staining to locate β-arrestin-2 and GPR182. Scale bars: 10 μm. Data shown are representative of 3 independent experiments. (**D**) GPR182^+^ HEK293T and control cells were examined by flow cytometry for Dil-HDL binding. GAG-bp was added to assess its blocking capacity. (**E**) GPR182^+^ HEK293T and control cells were examined by flow cytometry for Dil-LDL binding. GAG-bp was added to assess its blocking capacity. (**F**) GPR182^+^ HEK293T cells were stained for Dil-labeled lipoproteins at 4°C. Cells were preincubated with GPR182 mAb (clone 1A5) at different concentrations to assess its blocking capacity. (**G**, **H**) GPR182^+^ cells (**G**) or ACKR2^+^ cells (**H**) were stained for CXCL10-AF647 at 4°C. Cells were preincubated with different concentrations of lipoproteins to assess their blocking capacities. Data represent the mean ± SEM.

Our previous study revealed that GPR182 is an ACKR that interacts with chemokines via the GAG-binding motif (16). Many apolipoproteins or lipoproteins are known to interact with GAGs (26, 27). Given what we have observed in *Gpr182^–/–^* mice with diet-induced obesity, we hypothesized that GPR182 is a receptor on lymphatics that mediates lipoprotein transport. First, we confirmed by flow cytometry that apolipoprotein B-100 (ApoB-100) (Figure 3A) and apolipoprotein E (ApoE) (Figure 3B), two major ligand-binding apolipoproteins with known GAG-binding capacities (28), bound to human GPR182^+^ 293T cells. Inclusion of a mAb against GPR182 (clone 1A5) (Supplemental Figure 4A) markedly reduced the bindings (Figure 3, A and B), demonstrating the specific interaction between GPR182 and these 2 apolipoproteins.

GPR182 is a newly characterized ACKR that constitutively associates with β-arrestin-2 (15–17). In a NanoBit assay, cotransfection of GPR182 and β-arrestin-2 in HEK293T cells triggered a strong interaction signal (16). The addition of human serum at 1% was able to effectively abolish the signal (Supplemental Figure 4B). Similarly, all 4 major serum lipoproteins inhibited the interaction between GPR182 and β-arrestin-2 in a dose-dependent manner (Supplemental Figure 4C). Among them, VLDL and CM, were the 2 most effective lipoproteins at disrupting the GPR182–β-arrestin-2 association, in comparison with HDL and LDL. We induced the expression of β-arrestin-2–EGFP in GPR182^+^ and control CHO cells to better visualize the interaction between β2-arrestin and GPR182. We found that β-arrestin-2 was homogenously distributed within the cytoplasm of WT CHO cells, whereas β-arrestin-2-2 in GPR182^+^ CHO cells tended to aggregate with GPR182 (Figure 3C). Interestingly, the addition of all 4 lipoproteins in GPR182^+^ CHO cells effectively disrupted the aggregation between GPR182 and β-arrestin-2 (Figure 3C).

We directly examined whether lipoproteins from human serum interact with GPR182. At 4°C, we found that Dil-HDL had stronger binding to GPR182^+^ HEK293T cells compared with WT HEK293T cells, and this binding was abolished by a generic GAG-binding peptide (GAG-bp) (Figure 3D) (16). Similarly, Dil-LDL bound to GPR182^+^ HEK293T cells, and this interaction was competed off by GAG-bp (Figure 3E). All 4 major serum lipoproteins bound to GPR182-expressing cells, which was effectively blocked by the GPR182 mAb (clone 1A5) (Figure 3F). The same GPR182 mAb recognized both human and mouse GPR182 with high affinity and was able to completely block chemokine CXCL10 binding (Supplemental Figure 4, D and E). We found that all 4 lipoproteins competed with CXCL10 for GPR182 binding, further supporting the idea of an overlap in binding sites between chemokines and lipoproteins on GPR182 (Figure 3G). Interestingly, the same lipoproteins did not interfere with CXCL10 binding to ACKR2 (Figure 3H), another ACKR that CXCL10 interacts with (29). All these observations indicate that GPR182 specifically interacted with lipoproteins in vitro.

### GPR182 mediates lipoprotein transport.

**Figure 4:**
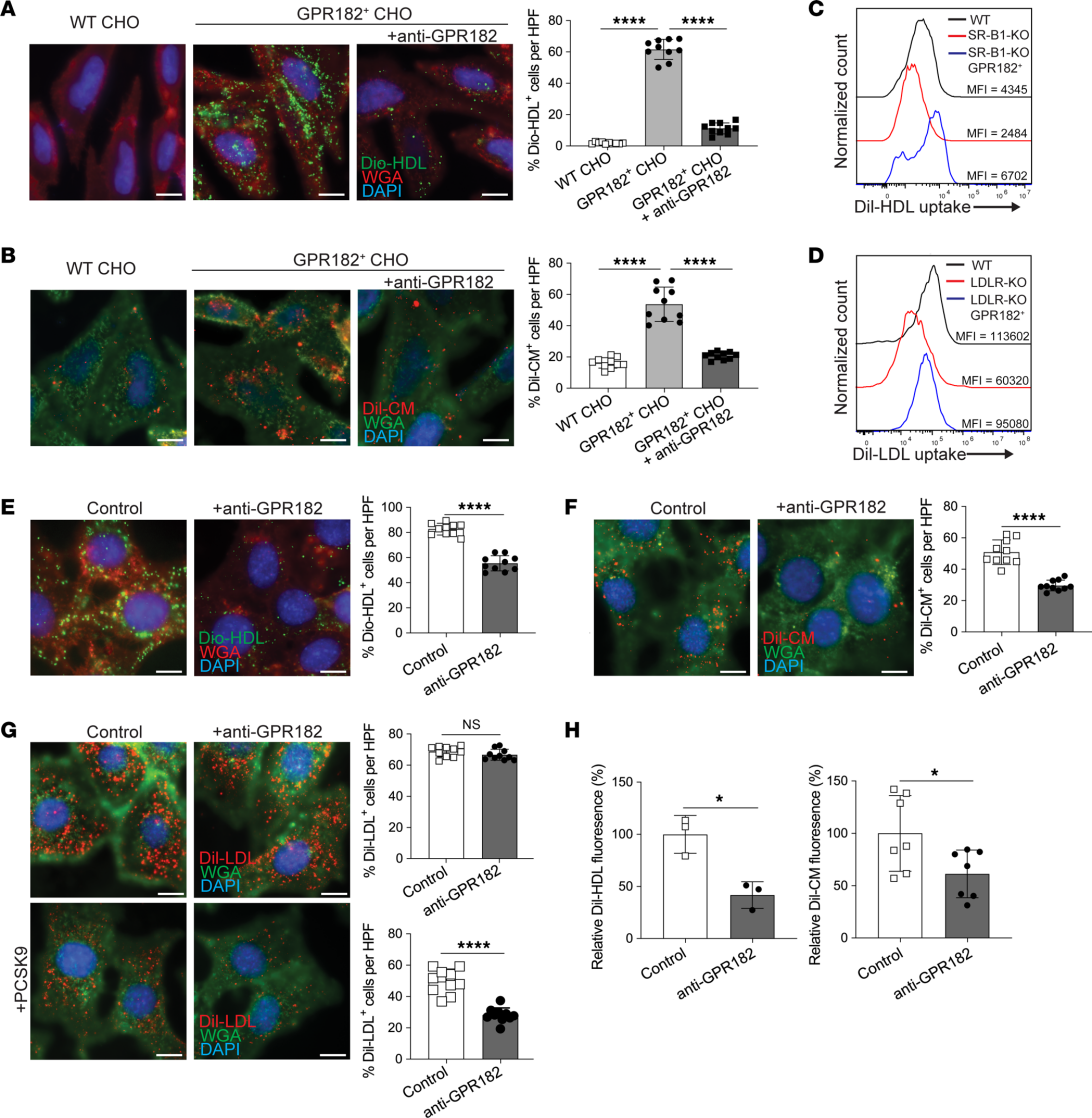
GPR182 mediates lipoprotein transport. (**A** and **B**) GPR182^+^ CHO and control WT CHO cells were incubated with Dio-HDL (**A**) or Dil-CM (**B**) at 37°C to assess lipoprotein endocytosis. GPR182 mAb (clone 1A5) was added to assess its blocking capacity. Cells were costained with WGA before imaging. (**C**) Dil-HDL uptake was assessed in WT HEK293T cells, SR-B1-KO cells, and SR-B1–KO cells transfected with GPR182 at 37°C. (**D**) Dil-LDL uptake was assessed in WT HEK293T cells, LDLR-KO cells, and LDLR-KO cells transfected with GPR182 at 37°C. (**E** and **F**) SVEC4-10 cells were treated with control or GPR182 mAb before incubation with Dio-HDL (**E**) or Dil-CM (**F**) at 37°C for 30 minutes. Cells were then costained with WGA to assess lipoprotein endocytosis. (**G**) SVEC4-10 cells, with or without PCSK9 treatment, were incubated with control or GPR182 mAb before culturing with Dil-LDL at 37°C for 30 minutes. Cells were then costained with WGA to assess lipoprotein endocytosis. (**H**) Dil-labeled HDL or CM was added into the inserts at 37°C. After overnight incubation, supernatant from the lower chambers was collected, and fluorescence was quantified using a fluorescence plate reader (*n* = 3 or 7). All experiments were repeated 2–3 times. Data represent the mean ± SEM. One-way ANOVA was used to compare the means of more than 2 groups (**A** and **B**). An unpaired, 2-tailed Student’s *t* test was used to compare the means of 2 groups (**E**–**H**). *P < 0.05 and ****P < 0.0001. Scale bars: 10 μm.

We further investigated the capacity of GPR182 to mediate lipoprotein endocytosis by incubating fluorescence-labeled lipoproteins with CHO cells at 37°C. GPR182^+^ CHO cells, but not control WT CHO cells, efficiently internalized HDL, and this uptake was completely inhibited by the GPR182 mAb (Figure 4A). Similarly, CM uptake by GPR182^+^ CHO cells was effectively suppressed by the anti-GPR182 mAb (Figure 4B).

LDL receptor (LDLR) and scavenger receptor class B type 1 (SR-B1) are the 2 known receptors for LDL and HDL, respectively (30–33). We assessed their involvement in lipoprotein uptake by GPR182 in HEK293T cells, which constitutively express LDLR and SR-B1 (Supplemental Figure 4, F and G). HEK293T cells effectively endocytosed Dil-HDL, presumably through SR-B1, as KO of SR-B1 in HEK293T cells (SR-B1–KO) abolished this uptake. Transfection of SR-B1–KO cells with GPR182 substantially increased HDL endocytosis (Figure 4C). Similarly, KO of LDLR in HEK293T cells limited their LDL uptake, but GPR182 transfection restored the cells’ capacity to internalize LDL (Figure 4D).

We used SVEC4-10, a murine LEC line (34), to further assess the involvement of endogenous GPR182 in lipoprotein uptake. Surface LDLR and SR-B1 were constitutively expressed in SVEC4-10 cells (Supplemental Figure 4H and S4I), while GPR182 protein was primarily present intracellularly (Supplemental Figure 4J). Preincubating SVEC4-10 cells with GPR182 mAb effectively blocked the cells’ uptake of HDL (Figure 4E) and CM (Figure 4F). SVEC4-10 endocytosed LDL vigorously, and GPR182 mAb alone was unable to inhibit LDL uptake. However, once SVEC4-10 cells were incubated with PCSK9 protein to reduce surface LDLR (Supplemental Figure 4K), inclusion of the GPR182 mAb was able to significantly reduce LDL endocytosis (Figure 4G).

The involvement of GPR182 in lipoprotein endocytosis led us to further assess its capacity to mediate lipoprotein transcytosis. Receptor-mediated lipoprotein transcytosis has previously been observed in cultured endothelial cells (35–38). We performed a Transwell assay to study whether GPR182 blockade affects lipoprotein transport through a layer of LECs (35, 39). Dil-labeled HDL or CM was added to the upper chamber of a confluent layer of SVEC4-10 cells cultured on inserts at 37°C. After overnight incubation, quantification with a fluorescence plate reader in the lower chamber revealed that preincubation with the GPR182 mAb significantly reduced the transfer of both lipoproteins (Figure 4H), which was further verified by fluorescence imaging (Supplemental Figure 4, L and M). These results collectively point to GPR182 as an independent receptor that mediates lipoprotein uptake and transport.

### GPR182 regulates lipoprotein homeostasis.

The broad expression of GPR182 led us to assess the possible effect of GPR182 ablation on systemic lipoprotein homeostasis. *Gpr182^–/–^* mice on a regular chow diet had markedly higher total serum cholesterol levels than did age-matched WT mice (Supplemental Table 1). Cholesteryl ester, but not free cholesterol, was increased in the serum of *Gpr182^–/–^* mice. In contrast, serum TAG and FFAs were slightly reduced in *Gpr182^–/–^* mice. All these changes were consistently found in both female and male *Gpr182^–/–^* mice at different ages (Supplemental Table 1). Fast protein liquid chromatography (FPLC) analysis further revealed a selective increase of serum HDL cholesterol levels in *Gpr182^–/–^* mice (Supplemental Figure 5, A and B). Mass spectrometric analysis of serum proteins from *Gpr182^–/–^* mice showed an increase in expression levels of multiple apolipoproteins, including apolipoprotein C-IV, apolipoprotein D, apolipoprotein A-II, apolipoprotein B-100, and apolipoprotein C-I (Supplemental Figure 5C) (40). Our results thus show that GPR182 contributed to lipoprotein homeostasis in vivo.

### GPR182 on lacteals mediates CM transport.

**Figure 5:**
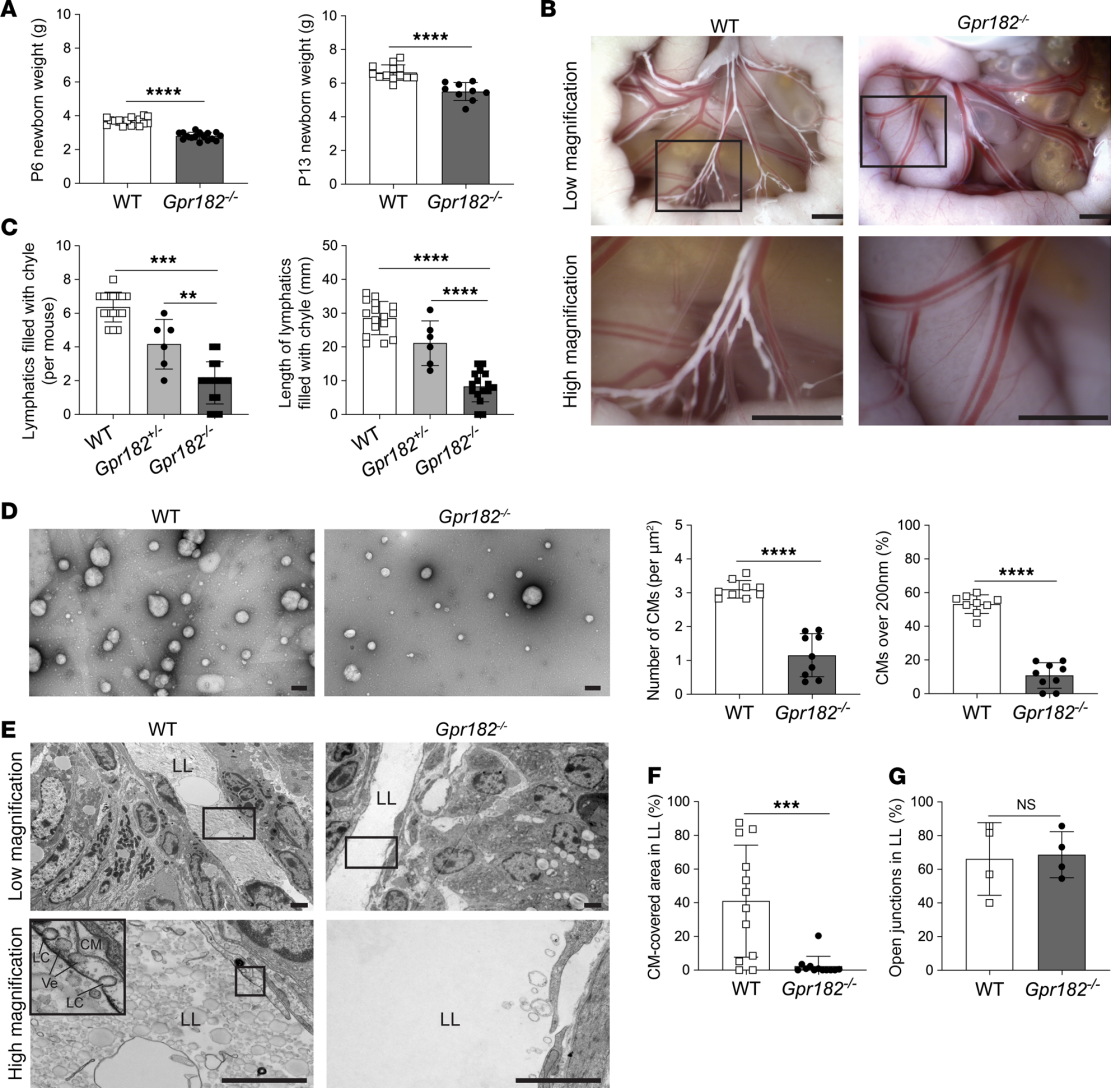
Limited CM transport in *Gpr182^–/–^* lacteals. (**A**) BWs of *Gpr182^–/–^* mice and their littermates at P6 (*n* = 16) and P13 (*n* = 9, 13) were measured. Data were pooled from 3 independent experiments. (**B** and **C**) P6 newborns of *Gpr182^–/–^* mice and their littermates were examined for mesenteric lymphatics. Representative images (**B**) and quantification (**C**) of lymphatics filled with CMs. Each symbol represents 1 mouse. Scale bars: 1 mm. Data were pooled from 4 independent experiments. (**D**) TEM analysis of mesenteric lymph fluid for CMs from P6 newborns of WT and *Gpr182^–/–^* mice. The number and size of CMs were quantified. Each symbol represents 1 field (*n* = 3 per group). Scale bars: 200 nm. (**E**) TEM analysis of intestinal villus from adult female WT and *Gpr182^–/–^* mice 2 hours after olive oil gavage. Note that large caveola and lipoprotein-containing vesicles were only found in LECs of WT mice. LL, lacteal lumen; LC, large caveola; Ve, vesicle containing lipoproteins. Scale bars: 5 μm. Quantification of CMs in intestinal lacteal lumens (**F**) and open lacteal junctions (**G**) via TEM in adult mice. Each symbol represents 1 lacteal lumen (**F**) or 1 mouse (**G**) (*n* = 4 per group). Data were pooled from 2 independent experiments and represent the mean ± SEM. An unpaired, 2-tailed Student’s *t* test was used to compare the means of 2 groups (**A**, **D**, **F**, and **G**). One-way ANOVA was used to compare the mean of more than 2 groups (**C**). **P < 0.01, ***P < 0.001, and ****P < 0.0001.

We further confirmed the involvement of GPR182 in lipid absorption by analyzing newborns during the lactation period. *Gpr182^–/–^* pups were visually indistinguishable from WT littermates (Supplemental Figure 6A). However, they weighed slightly, but significantly, less than their WT littermates throughout the course of lactation (Figure 5A). Collecting mesenteric lymphatics from 7-day-old (P6) *Gpr182^–/–^* newborns were pale or transparent, whereas those from their WT counterparts appeared milky-white (Figure 5B). Quantification of lymphatic collectors in P6 pups revealed that *Gpr182^–/–^* pups contained far fewer collecting lacteals filled with white CMs than did WT controls (Figure 5C). TEM analysis confirmed a remarkable reduction of CM particles in mesenteric lymph fluid from *Gpr182^–/–^* newborns (Figure 5D).

We performed TEM to visualize CM transport in lacteals. Two hours after oral oil gavage in adult mice, lipid droplets and CMs in enterocytes were abundant in both WT and *Gpr182^–/–^* mice (Supplemental Figure 6B). Few CMs were present in the lacteal lumens of *Gpr182^–/–^* mice, in sharp contrast to the lacteal lumens of WT mice, which were full of CM particles (Figure 5E). Quantification analysis further confirmed a sharp reduction of CMs in the lacteal lumens of *Gpr182^–/–^* mice (Figure 5F). It was only in the intestines of WT mice, upon oil gavage, that we observed many lipoprotein-containing vesicles in LECs and CMs budding from LECs into the lacteal lumen (Figure 5E), indicating a transcytosis mechanism for CM transport. There was no structural alteration in junction openness of lacteal lumens in *Gpr182^–/–^* mice (Figure 5G). In newborn pups under breastfeeding, lacteal lumens from WT newborns were abundantly filled with CMs, whereas CMs were scarce in lacteals from *Gpr182^–/–^* pups (Supplemental Figure 6C). However, junction openness in lacteal lumens was similar between WT and *Gpr182^–/–^* pups (Supplemental Figure 6C). Together, our results support the notion that GPR182 is required for CM transport in the small intestine.

Besides LECs, GPR182 is expressed by microvascular and sinusoidal ECs across multiple organs (15, 19, 41). To verify the involvement of lymphatic GPR182 in lipid transport, we generated lymphatics-specific GPR182-KO mice by crossing *Gpr182*^fl/fl^ mice with Lyve1-Cre–transgenic mice. We confirmed the selective deletion of GPR182 in lymphatics by flow cytometry (Supplemental Figure 7A). Like global *GPR182*-KO mice, *Gpr182*^LEC-KO^ mice were markedly protected from HFD-induced obesity, characterized by reduced BW gain and fat mass compared with littermate controls (Supplemental Figure 7, B–E). These findings provide additional evidence supporting a key role for lacteal GPR182 in mediating intestinal CM absorption.

### GPR182 blockade limits dietary lipid absorption in HFD-induced obesity.

**Figure 6:**
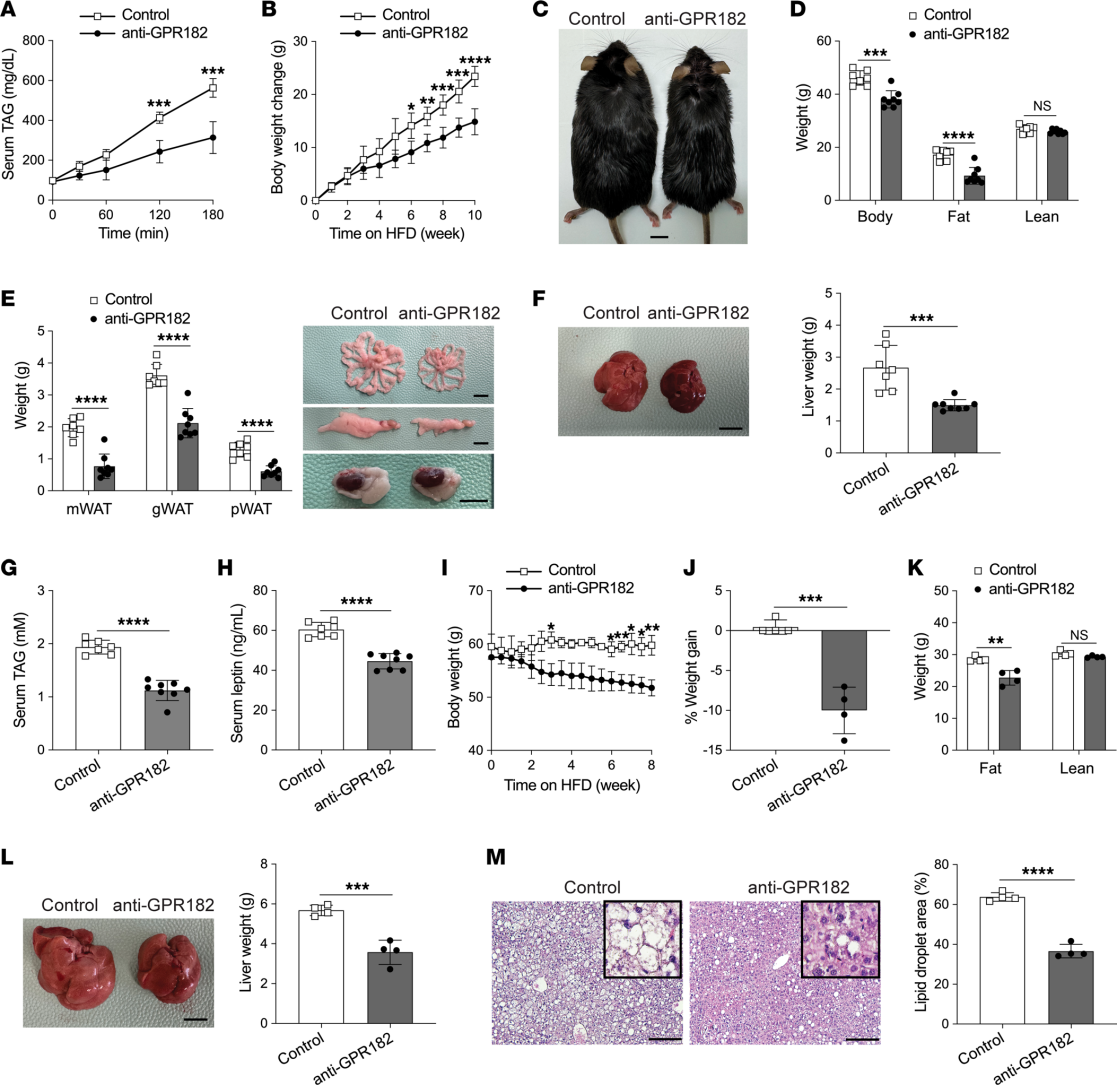
GPR182 blockade effectively treats HFD-induced obesity. (**A**) Female WT B6 mice pretreated with control or anti-GPR182 mAb (clone 1A5) were assessed for serum TAG levels upon olive oil gavage (*n* = 7). (**B**–**H**) Seven-week-old male WT B6 mice were fed a HFD for 10 weeks and treated weekly with control or anti-GPR182 mAb from the start of HFD feeding. Data shown are representative of 3 independent experiments. (**B**) BW gain was followed weekly (*n* = 7, 8). (**C**) Representative image of mice after 10 weeks of HFD feeding (scale bar: 1 cm). (**D**) BW, fat, and lean mass were recorded. (**E**) The weights of WATs, including mWAT, gWAT, and pWAT, were calculated. Representative images are shown (scale bars: 1 cm). (**F**) Representative images of livers (scale bar: 1 cm). Liver weights were calculated. Serum TAG (**G**) and leptin (**H**) levels after 10 weeks of a HFD were determined. (**I**–**M**) Male DIO WT B6 mice were treated with control or anti-GPR182 mAb (clone 1A5) twice weekly for 8 weeks (*n* = 4). (**I**) BW was assessed twice weekly. (**J**) BW changes were determined after 8 weeks of treatment. (**K**) Fat and lean masses were determined by MRI. (**L**) Representative liver images (scale bar: 1 cm) and liver weights. (**M**) Images of H&E-stained livers (scale bars: 200 μm; insets, original magnification: ×400) and quantification of lipid droplet area. Data represent the mean ± SEM. Two-way ANOVA with Bonferroni’s correction for multiple comparison was used to compare groups over time by repeated measures (**A**, **B** and **I**). An unpaired, 2-tailed Student’s *t* test was used to compare the means of 2 groups (**D**–**H**, and **J**–**M**). *P < 0.05, **P < 0.01, ***P < 0.001, and ****P < 0.0001.

We assessed whether blockade of GPR182 can reduce dietary lipid absorption and therefore attenuate HFD-induced obesity. To do this, we took advantage of the strong cross-binding to mouse GPR182 for the mAb clone 1A5 (Supplemental Figure 4E), which effectively disrupted the lipoprotein-GPR182 interaction (Figures 3 and 4). Mice treated with 1A5 lowered serum TAG upon olive oil gavage (Figure 6A). Weekly treatment with GPR182 mAb did not have an instant effect on weight gain in response to a HFD. However, after 3 weeks of HFD feeding, we began to observe a weight gain difference between these 2 mouse groups (Figure 6B). Following 10 weeks of a HFD, the average BW of the control mice was 46 g, while mice treated with GPR182 mAb weighed an average of 38 g (Figure 6, C and D). The reduced weight gain of GPR182 mAb–treated mice was primarily due to lower fat mass (Figure 6D). Fat tissues such as mesenteric WAT (mWAT), gonadal WAT (gWAT), and perirenal WAT (pWAT) from mice treated with GPR182 mAb consistently weighed significantly less than did tissues from control mice (Figure 6E). Treatment with 1A5 in HFD-challenged mice led to a marked reduction in liver weight (Figure 6F). Serum levels of TAG (Figure 6G) and leptin (Figure 6H) were significantly lower in mice treated with GPR182 mAb.

We found similar anti-obesity effects in human GPR182-knockin (h*Gpr182*-KI) mice when we used clone 11C7 (Supplemental Figure 4A), another GPR182-blocking mAb that only recognizes human GPR182 (Supplemental Figure 8A). Administration of 11C7 weekly to h*Gpr182*-KI mice alleviated HFD-induced obesity and its associated liver steatosis (Supplemental Figure 8, B–G). In addition, h*Gpr182*-KI mice treated with 11C7 produced more feces (Supplemental Figure 8H) that contained more lipid residues (Supplemental Figure 8I). All these findings were consistent with what we observed in *Gpr182^–/–^* mice on a HFD.

We investigated whether GPR182 blockade could serve as a therapeutic strategy for existing obesity. Diet-induced obese (DIO) WT B6 mice were treated twice a week with either a control or 1A5 mAb while maintained on a HFD throughout the treatment period. Anti-GPR182 treatment led to a gradual reduction in BW, which was noticeable as early as week 2 (Figure 6I). After 8 weeks, mice receiving the GPR182 mAb lost 10% of their BW, whereas control mice maintained their initial weight (Figure 6J). MRI analysis revealed that anti-GPR182 mAb specifically reduced total fat mass with minimal effect on lean mass (Figure 6K). Consistently, mice on therapy had significantly smaller WATs (Supplemental Figure 9, A–C). Additionally, GPR182 mAb markedly alleviated hepatic steatosis in obese mice (Figure 6, L and M). Collectively, these findings support GPR182 blockade as a promising approach to treat obesity and to improve existing anti-obesity therapies.

## Discussion

We demonstrated that GPR182 serves as a lipoprotein receptor that mediates fat absorption in the small intestine. This finding is based on our studies of Gpr182–/– mice and experiments using 2 different GPR182-blocking mAbs. Our findings thus provide direct molecular evidence to support a receptor-mediated transcytosis mechanism for dietary fat absorption. While glucagon-like peptide 1 (GLP-1) agonists remain the standard treatment for obesity (42–44), GPR182 blockade offers a distinct, fat-targeted mechanism that selectively reduces adipose tissue while sparing lean mass — an advantage that may improve body composition and complement existing therapies.

Lacteals in the small intestine are known to mediate lipid absorption in the form of CMs. CMs have been thought to be transported paracellularly as the discontinuous, button-like junctions in lymphatic capillaries allow CMs to cross (45). However, the few large open junctions in lacteals observed by early TEM imaging would argue against the role of these junctions as the primary route for CM transport into the lacteals (12, 46). Vesicles containing lipoprotein particles and large caveolae have been found in lacteal LECs of mice upon oral oil gavage, supporting a role of transcellular transport in lipid absorption (11). Furthermore, it was reported that, in an in vitro model, the transport of lipids across the lymphatic endothelium occurs via an ATP-dependent, transcellular route (14). Our results indicate receptor-mediated transcytosis as the primary mechanism for CM transport in the small intestine. *Gpr182^–/–^* mice showed no alteration in lacteal structure and had no defect in CM synthesis in the small intestine. However, loss of GPR182 led to a dramatic reduction in CMs crossing into lacteal lumens in the small intestine. As a result, *Gpr182^–/–^* mice under HFD feeding or treated by oral oil gavage displayed lipid droplet accumulation in the small intestine. Furthermore, HFD-fed *Gpr182^–/–^* mice had larger amounts of fecal lipids. *Gpr182^–/–^* pups exhibit retarded weight gain during breastfeeding, and adult *Gpr182^–/–^* mice are resistant to HFD-induced obesity. This protection is not caused by a defect in lymphatic function, as lymphatic draining in *Gpr182^–/–^* mice appears to be normal (16). Furthermore, the application of 2 different GPR182-blocking mAbs in a HFD-induced obesity model fully recapitulates what we observed in *Gpr182^–/–^* mice. All these findings support a major role of GPR182 in mediating dietary lipid absorption. On the other hand, our study does not negate the involvement of free CM transport through open cell-to-cell junctions between LECs within the lacteals. *Gpr182^–/–^* mice possess normal button-like junctions, which would permit normal paracellular lipid entry. Consistently, we found residual CMs in lacteals of *Gpr182^–/–^* mice. In addition, some mesenteric connecting lymphatics from *Gpr182^–/–^* newborns were filled with CMs. It is certainly possible that GPR182 is not the only receptor to mediate CM transcytosis. There is also a possibility that the presence of GPR182 facilitates paracellular lipid transport via its interaction with CMs. The respective contribution in relation to these 2 different processes for fat absorption would require careful comparison in different biological conditions.

GPR182, recently named ACKR5 (17, 47), belongs to a group of GPCRs that primarily function as chemokine scavengers (48). However, the known ligands for ACKRs are not exclusively chemokines. For example, ACKR3, also called CXCR7, is a receptor for adrenomedullin (49) and a broad scavenger of opioid peptides (50, 51). CCRL2 and GPR1, which are 2 new members of ACKRs, bind chemerin, a non-chemokine attractant (48). Our previous study identified GPR182 as a pattern recognition receptor for GAG-binding peptides (16). Similar to chemokines, apolipoproteins such as ApoB and ApoE are known to be GAG-interacting proteins (26, 52). Here, we were able to confirm their GPR182 binding and found that lipoproteins and chemokines shared a similar binding site on GPR182. Compared with most chemokines, lipoproteins have weaker binding to GPR182 (15, 16, 20). This is consistent with their much higher abundance in the body, suggesting that weaker binding may help prevent receptor saturation, while allowing continuous interaction. Supporting this finding, a recent report revealed that GPR182 interacts with many non-chemokine proteins (20). In a high-throughput screening assay, GPR182 was identified as 1 of 3 candidates that are potential binding receptors for LDL (37). Therefore, our characterization of GPR182 as a lipoprotein receptor is consistent with and extends these earlier findings.

The broad expression of GPR182 across diverse endothelial cell types supports its involvement in both exogenous fat absorption and endogenous lipid transport. Systemic lipoprotein levels are the outcome of a tightly regulated, dynamic balance between these 2 pathways (53). Although GPR182 serves as a receptor for multiple lipoproteins, *Gpr182^–/–^* mice on a regular chow diet are generally normal and only have higher levels of circulating HDL. In support of this, a GWAS showed that a GPR182 gene variant (rs58298943-C) is significantly associated with high serum HDL levels (*P* value: 4 × 10^–9^) (54). The presence of other specialized lipoprotein receptors could play a complementary role to compensate for the loss of GPR182 (30, 55). It would be interesting to further understand how GPR182 coordinates with other lipoprotein receptors to regulate lipoprotein transport and metabolism in various pathological conditions.

In conclusion, our studies identify a lipoprotein receptor in GPR182 that mediates lipid absorption in the small intestine. These findings establish a mechanistic link between GPR182 activity and dietary fat uptake. Agents that block GPR182-mediated lipoprotein transport may offer a promising therapeutic approach for treating obesity and other disorders related to lipid metabolism, potentially complementing existing strategies that primarily target appetite or energy expenditure.

## Methods

### Sex as a biological variable.

Our study of HFD-induced obesity exclusively examined male C57BL/6J mice because female mice are resistant to developing obesity. For other experiments, including oral oil gavage, serum lipoprotein profiling, and intestinal draining function measurement, both male and female mice were used with consistent results. We have explicitly indicated the sex of animals used for each experiment in the figure legends.

### Mice.

*Gpr182^–/–^* mice were generated by crossing *Gpr182^lacZ/lacZ^* mice (16) with CMV-Cre mice from The Jackson Laboratory (strain no. 006054). Genotyping for *Gpr182^–/–^* mice was performed with the following primers: forward, GCTACCATTACCAGTTGGTCTGGTGTC; reverse, AGAGAAAGGTCATCTGTGAGGAGGC. h*Gpr182*-KI mice were generated by Leveragen with the CRISPR/Cas9 technology, by replacing the coding sequence of mouse *Gpr182* with human *Gpr182* through homologous recombination. LEC-specific *GPR182*-KO mice (*Gpr182*^LEC-KO^) were generated by crossing *Gpr182*^fl/fl^ mice (strain no. T021071, GemPharmatech) with Lyve1-Cre–transgenic mice (56) (gift from Beth Tamburini, CU AMC, Aurora, Colorado, USA). Littermate *Gpr182*^fl/fl^ mice lacking Cre were used as controls. All mice were backcrossed with C57BL/6J mice on a background and were housed in a pathogen-free vivarium with a 14-hour light/10-hour dark cycle at the CU AMC. C57BL/6J mice purchased from The Jackson laboratory were housed at the CU AMC animal facility for at least 2 weeks before their use in experiments.

### Abs.

mAbs against human GPR182 were generated by hybridoma fusion of splenocytes from immunized *Gpr182^–/–^* mice (57). Antibody in culture medium was purified with a Protein G HP Column (Cytiva HiTrap). The specificity of GPR182 mAbs, including clones 1A5 and 11C7, was confirmed by their specific binding to GPR182-transfected cells using flow cytometry. The blocking capacity was further assessed by their effect on blocking the binding between GPR182^+^ cells and CXCL10-AF647 (ALMAC) and Dil-HDL (Kalen Biomedical).

### HFD-induced obesity.

Male WT and *Gpr182^–/–^* mice at 7 weeks of age were fed a rodent diet with 58 kcal percentage of fat and sucrose (Research Diets: D12331) for 16 weeks. Carbohydrates (42 g/L) were mixed in drinking water at a ratio of 55% fructose to 45% sucrose by weight (MilliporeSigma). Mice had ad libitum access to food and water and were weighed weekly. Similar procedures were used for GPR182 mAb administration. Male WT B6 or h*Gpr182*-KI mice at 7 weeks of age were injected i.p. with a control or anti-GPR182 mAb (clone 1A5 for WT B6, 11C7 for h*Gpr182*-KI) at 300 μg/mouse weekly. Mouse weights were measured weekly. For the treatment of existing obesity, DIO male WT B6 mice were treated with control or anti-GPR182 mAb (clone 1A5) twice a week at 300 μg/mouse, and mouse weights were measured twice a week.

### Energy balance assessment.

Energy balance assessment for *Gpr182^–/–^* and control WT mice under a HFD was conducted at the Energy Balance Assessment Core of the Colorado Nutrition Obesity Research Center (CNORC) (Aurora, Colorado, USA). Total energy expenditure (TEE), resting energy expenditure (REE), and nonresting energy expenditure (NREE) were measured in Oxymax chambers in a comprehensive laboratory animal monitoring system (CLAMS) (Columbus Instruments). Mice were given 1 week to become accustomed to a HFD before being placed individually in metabolic chambers with an animal activity meter (Opto-Max, Columbus Instruments) for 2 weeks of a HFD. Mice had ad libitum access to food and water. Metabolic chambers were maintained at 23°C with 12-hour light/12-hour dark cycles. The metabolic rate (MR) was calculated from gas exchange measurements acquired every 18 minutes using the Weir equation (58). Then, the MR was averaged and extrapolated over 24 hours to estimate the TEE. Before and after the energy balance assessment, whole-body fat and lean composition were measured by Echo MRI (Echo Medical Systems).

### Lipoprotein, TAG, lipids, and FFA quantification.

Blood samples were centrifuged at 1,000*g* for 10 minutes at 4°C to extract the plasma. The concentrations of cholesterol, TAG, FFA, and glucose in plasma samples were measured following the manufacturer’s instructions for the colorimetric assay kits (Merck-Millipore). The levels of leptin and insulin in plasma samples were quantified with ELISA kits (Merck-Millipore).

Lipids in mouse tissues were extracted using the Folch method (59). Fecal lipids were extracted using a modified Folch method (60). Briefly, samples were homogenized in chloroform in methanol (2:1 by volume) and centrifuged at 1,000*g* for 10 minutes. The supernatants were then collected, dried, and weighed. Finally, the extracts were redissolved in ethanol to measure lipids with the assay kits as described above.

Plasma samples (200 μL) were chromatographed via FPLC using 2 Superose 6 columns in series as previously reported (61). During size exclusion, the absorbance of the eluted samples was measured at 280 nm, allowing identification of each lipoprotein class via protein content. Fractions containing either VLDL, LDL, or HDL were pooled, and cholesterol was measured using a commercially available kit (Cayman Chemical) following the manufacturer’s instructions.

### Oil gavage assay.

After 12 hours of fasting and 30 minutes of tyloxapol pretreatment i.p. (500 mg/kg BW, Merck-Millipore), *Gpr182^–/–^* and control WT mice at the same age were given olive oil by oral gavage (10 μL/g BW). Blood samples were collected via the lateral tail vein before (time 0) and at 30, 60, 120, and 240 minutes after oil gavage. The levels of TAG in plasma samples were measured as described above. In some experiments, mice were pretreated with GPR182 mAb at 300 μg per mouse before oil gavage. Liver and intestine samples were collected 2 hours after oil gavage for H&E and Oil Red O staining. BODIPY FL C_16_ (Thermo Fisher Scientific, D3821) was added to the olive oil to make a final concentration of 0.4 μg/μL. After 6 hours of fasting, 2-week-old *Gpr182^–/–^* and control WT mice were orally administered diluted BODIPY FL C_16_ at 50 μL per mouse. Mice were euthanized 2 hours after the gavage. The whole intestine along with mesenteric lymphatics were put into a 35 mm glass-bottomed culture dish and imaged with a Zeiss Axio Observer.

### NanoBit assay.

HEK293T cells were cotransfected with 2 plasmids that encode for GPR182 tagged with LgBit and for β2-arrestin tagged with SmBit (16). Twenty-four hours later, cells were replated and incubated with lipoproteins at different concentrations. NanoGLO substrate was added right before luminescence measurement.

### Lipoprotein binding, endocytosis, and transcytosis in vitro.

For lipoprotein binding studies, both WT control and GPR182^+^ HEK293T cells were incubated with or without pretreatment with 1 mg/mL GAG-binding peptide at 4°C for 15 minutes. Cells were then incubated with Dil-LDL or Dil-HDL (Kalen Biomedical) at 4°C for 30 minutes. After cells were washed with cold PBS, flow cytometry was performed with the CytoFLEX (Beckman Coulter) to assess the binding of lipoproteins to GPR182-expressing cells. FlowJo 11 was used to calculate the MFI, and GraphPad Prism 9.0 (GraphPad Software) was used to analyze nonlinear regression. Fluorescence-labeled ApoB-100 or biotin-labeled ApoE protein was used to assess binding to GPR182^+^ HEK293T cells, and GPR182 mAb (clone 1A5) was added to validate the involvement of GPR182. For lipoprotein endocytosis, WT CHO (ATCC, CCL-61) and GPR182^+^ CHO cells were serum starved for 6 hours before incubation with the lipoproteins Dio-HDL (Kalen Biomedical, LLC, 10 μg/mL) or Dil-CM (10 μg/mL) at 37°C for 30 minutes. GPR182 mAb (clone 1A5) was added to assess the involvement of GPR182. Cells were stained with wheat germ agglutinin (WGA) (Thermo Fisher Scientific) and spectral DAPI (Akoya Biosciences) before analysis. CMs (Medix Biochemica) were labeled with Dil (Thermo Fisher Scientific, 20 μg Dil/mg CM) by incubation at 37°C overnight, and excess dye was removed by 3 rounds of dialysis against PBS. In some experiments, SVEC4-10 cells (ATCC-CRL-2161, gift from Beth Tamburini, UC AMC, Aurora Colorado, USA) were used to evaluate lipoprotein endocytosis (Dio-HDL, Dil-CM, Dil-LDL, 10 μg/mL, at 37°C for 30 minutes). GPR182 mAb (clone 1A5) was added to assess the involvement of GPR182. Images were taken with the Zeiss Axio Observer. For lipoprotein transcytosis, SVEC4-10 cells were seeded onto TC-treated, 0.4 μm pore size Transwell inserts (Corning). An initial seeding was followed by a second seeding 12 hours later. A confluent layer was confirmed by microscopy and permeability assay with 500 kDa dextran (tetramethylrhodamine isothiocyanate–dextran [TRITC-dextran], MilliporeSigma, catalog 52194). Dil-HDL (10 μg/mL) or Dil-CM (50 μg/mL) was added to the Transwell inserts, and supernatant from the lower chambers was collected after overnight incubation. Fluorescence in the lower chamber supernatant was quantified using a fluorescence plate reader, and representative fluorescence images were acquired for visualization only. GPR182 mAb (clone 1A5) was added to assess the involvement of GPR182.

### IHC and immunofluorescence staining.

For H&E staining, tissues were formalin fixed and embedded in paraffin. The blocks were sectioned at 5 μm and stained with H&E. For Oil Red O staining, tissues were frozen on dry ice in OCT embedding media. Then, the blocks were sectioned at 8 μm and stained with Oil Red O, followed by counterstaining with hematoxylin. For immunofluorescence staining of tissues, paraffin blocks (5 μm) were subjected to deparaffinization, rehydration, and antigen retrieval; OCT-embedded frozen sections (8 μm) were washed to remove OCT and directly blocked prior to staining. After blocking with the appropriate normal serum (from the host species of the secondary Ab), slides were incubated with the appropriate primary Abs overnight at 4°C. After washing 3 times with 1× PBS, the slides were stained with fluorophore dye–conjugated secondary Abs for 2 hours and DAPI for 10 minutes at room temperature (RT). Slides were cleared and mounted with Fluoromount-G Mounting Medium (Thermo Fisher Scientific) before imaging with the Zeiss Axio Observer. Sometimes images were taken with the Olympus FV1000 FCS confocal laser-scanning microscope. For immunofluorescence staining, cells were seeded onto poly-d-lysine–pretreated (Thermo Fisher Scientific) 12 mm glass slides in a 24-well plate. The glass slides with cells were subjected to 4% paraformaldehyde (PFA) fixation, permeabilization, and blocking with the appropriate normal serum. Cells were then incubated with fluorophore dye–conjugated primary Abs overnight at 4°C. After washing, cells were stained with DAPI for 10 minutes at RT. Slides were cleared and mounted with Fluoromount-G Mounting Medium (Thermo Fisher Scientific) before imaging with the Zeiss Axio Observer. Small intestines of mice were harvested and cut longitudinally to expose the lumen. After washing the luminal contents with cold PBS, the intestines were pinned on stiff paperboard with the villus side up and fixed in 4% PFA for 2 hours at RT followed by 2 consecutive washes with PBS to remove any remaining PFA. Fixed intestines were then embedded in 3% low-melt agarose (GoldBio) and sectioned with a vibratome (125 μm, 0.8 mm/s, 0.8 mm) to obtain sections with single rows of intestinal villi. Tissue sections were washed with ice-cold PBS 3 times and subsequently washed with 10% sucrose in PBS for 2 hours. Then, the sections were incubated with 20% sucrose and 10% glycerol in PBS overnight at 4°C, followed by blocking with 5% donkey serum in 0.3% Triton X-100 (Thermo Fisher Scientific) in PBS for 2 hours at RT. The intestine sections were then incubated with primary Abs diluted in blocking solution for 24 hours at 4°C. After washing with ice-cold PBS, the sections were incubated with secondary Abs in blocking solution for 12 hours at 4°C, followed by DAPI incubation for 30 minutes at RT. Intestine sections were then washed with PBS and mounted with Fluoromount-G Mounting Medium before imaging with the Zeiss Axio Observer. Images were analyzed using ImageJ (NIH). The main primary Abs used in this study are as follows: anti–human GPR182 (catalog FAB10293R, R&D Systems); anti–mouse LYVE-1 (catalog AF2125, R&D Systems; catalog 11-034, AngioBio); anti–mouse CD31 (catalog AF3628, R&D Systems); anti–mouse VE-cadherin (catalog AF1002, R&D Systems); and anti–mouse Ki-67 (catalog 652402, BioLegend).

### TEM.

Jejunum tissues were dissected from mice after transcardiac perfusion with saline followed by 4% PFA and 1% glutaraldehyde in 0.1 M sodium cacodylate buffer. Samples were then fixed overnight with 2.5% (v/v) glutaraldehyde in 0.1 M sodium cacodylate buffer. After 5 rinses with 0.1 M sodium cacodylate buffer, tissues were postfixed in 1% osmium tetroxide and 0.8% potassium ferrocyanide in 0.1 M sodium cacodylate buffer for 1.5 hours. Tissue samples were rinsed 5 times in water and en bloc stained with 2% uranyl acetate in 50% ethanol for 2 hours. They were then dehydrated with increasing concentrations of ethanol, transitioned into resin with propylene oxide, infiltrated with EMbed-812 resin, and polymerized in a 60°C oven overnight. Blocks were sectioned with a diamond knife (Diatome) on a UC7 ultramicrotome (Leica) and collected onto copper grids and then poststained with 2% aqueous uranyl acetate and lead citrate. Images were acquired on a Tecnai T12 transmission electron microscope (Thermo Fisher Scientific) equipped with a LaB6 source at 120 kV using a XR80 (8 Mpix) camera (AMT Imaging).

### Negative stain electron microscopy.

Liquid was collected from the mesenteric lymph nodes (mLNs) of mice and diluted at 1:50 in PBS. A 10 μL drop of sample was applied to a freshly glow-discharged 300 mesh formvar and carbon-coated grid (Electron Microscopy Sciences) for 10 minutes and blotted with filter paper. The grid was then washed once by applying 10 μL water on the grid and blotted with Whatman filter paper. Finally, the grids were stained with 10 mL 2% uranyl acetate solution for 3 minutes. After blotting, the grids were allowed to dry. Samples were imaged on a Thermo Fisher Tecnai G2 Biotwin transmission electron microscope (Thermo Fisher Scientific) at 120 kV with an AMT low-mount NS15B sCMOS camera (AMT Imaging).

### Flow cytometric analysis.

Surface GPR182, LDLR, and SR-B1 expression analysis was performed by flow cytometry with CytoFLEX (Beckman Coulter). Briefly, cells were harvested, washed with ice-cold PBS, and resuspended in staining buffer (PBS containing 2% FBS). To block nonspecific binding, cells were incubated with Fc receptor–blocking Abs (anti-CD16/CD32) for 10 minutes on ice. Cells were then incubated with the appropriate staining Abs for 30 minutes at 4°C in the dark. Cells were washed twice with staining buffer, and flow cytometry analysis was performed. Data were analyzed with FlowJo 11 software. For LSEC isolation, mice were euthanized, and the inferior vena cava was cannulated. The liver was first perfused with PBS, followed by perfusion buffer over 5–10 minutes (DNase I (Roche, 100 μg/mL), collagenase P (MilliporeSigma, 200 μg/mL), and dispase II (Roche, 800 μg/mL). The liver was excised and mechanically dissociated to prepare the cell suspension, which was further incubated at 37°C for 15 minutes in the same enzyme cocktail to complete digestion. Cells were then washed with FACS buffer, treated with ACK lysis buffer, and incubated with Fc-block prior to staining. Small intestines were isolated, and Peyer’s patches were removed. Tissues were incubated in PBS containing 2% FBS and DTT (Thermo Fisher Scientific, 1 mM) at 37°C for 20 minutes to remove mucus and epithelial debris, followed by incubation in PBS containing 2% FBS and EDTA (MilliporeSigma, 1.3 mM) at 37°C for 1 hour to detach epithelial cells. The remaining tissue was digested with collagenase IV (Thermo Fisher Scientific, 0.3 mg/mL) at 37°C for 1 hour to generate a single-cell suspension. Cells were then washed with FACS buffer, treated with ACK lysis buffer to remove erythrocytes, and incubated with Fc-block prior to staining. The main staining Abs used in this study are as follows: anti–human GPR182 (catalog FAB10293R, R&D Systems); anti–human LDLR (catalog 565653, BD Biosciences); anti–mouse LDLR (catalog FAB2255P, R&D Systems); anti–human SR-B1 (catalog 363203, BioLegend); anti–mouse SR-B1 (catalog 163102, BioLegend); anti–mouse CD45 (catalog 103122, BioLegend); anti–mouse CD146 (catalog 134704, BioLegend); Ghost Dye Red 780 (catalog 13-0865-T100, CYTEK); anti–mouse CD45 (catalog 103155, BioLegend); anti–mouse CD31 (catalog 102406, BioLegend); and anti–mouse Lyve1 (catalog FAB2125P, R&D Systems). Data were acquired on a CytoFLEX (Beckman Coulter) and analyzed using FlowJo 11 software.

### Western blotting.

The jejunum was dissected from mouse small intestine, opened longitudinally, and rinsed with cold PBS to remove luminal contents. Mucosal cells were lysed in M-PER mammalian protein extraction reagent (Thermo Fisher Scientific) containing protease and phosphatase inhibitors (Thermo Fisher Scientific). Lysates were centrifuged at 12,000*g* for 15 minutes at 4°C to collect the supernatant. The protein concentration was measured using a bicinchoninic acid (BCA) assay. Protein samples were mixed with SDS-PAGE loading buffer (Bio-Rad) and boiled at 95°C for 10 minutes before being loaded onto SDS-PAGE gels (Bio-Rad) for separation. Proteins were transferred onto PVDF membranes (Bio-Rad) and blocked with EveryBlot blocking buffer (Bio-Rad) for 15 minutes at RT. Membranes were incubated with primary Abs overnight at 4°C, followed by washing and incubating with HRP-conjugated secondary Abs. Signal detection was performed using Pierce ECL Western blotting substrate (Thermo Fisher Scientific) and visualized with an imaging system. The main primary Abs used in this study were as follows: anti–mouse ApoB (catalog 20578-1-AP, Proteintech); anti–mouse MTP (catalog 612022, BD Biosciences); anti–mouse DGAT2 (catalog sc-293211, Santa Cruz Biotechnology); and anti–mouse β-Actin (catalog 3700S, Cell Signaling Technology).

### Functional lymphatic flow.

The intestinal lymphatic draining function was assessed by FITC-Dextran injection of a Peyer’s patch, as described previously (62). Briefly, the abdominal cavity of anesthetized mice was opened with a midline incision of the peritoneum to expose the intestine and mesentery. FITC-Dextran (2,000 kDa; 20 μg) (MilliporeSigma, FD2000S) was injected into a Peyer’s patch in the ileum. Blood samples were collected via the lateral tail vein 0, 5, 15, 30 and 60 minutes after injection. Mice were kept warm, and the intestine and mesentery were kept moist throughout the process. Plasma fluorescence was measured using the BioTek Synergy H1 Plate Reader. The excitation wavelength at 495 nm and the emission at 519 nm were used to detect fluorescence.

### Statistics.

All the experiments were repeated independently. Data are presented as the mean ± SEM. An unpaired, 2-tailed Student’s *t* test was used to compare the means of 2 groups. One-way ANOVA was used to compare the means of more than 2 groups. Two-way ANOVA with Bonferroni’s correction for multiple comparisons was used to compare groups over time by repeated measures. Energy expenditure was analyzed by ANCOVA with BW as a covariate. *P* values of less than 0.05 were deemed to be significant. GraphPad Prism 9.0 (GraphPad Software) was used for all statistical analysis and to generate figures.

### Study approval.

All animal experiments were approved by the IACUC at CU AMC.

### Data and materials availability.

All data associated with this study are present in the article or the supplemental materials. Abs for GPR182 are available from the UC AMC upon completion of a material transfer agreement. Values for all data points in graphs are reported in the Supporting Data Values file. The mass spectrometric proteomics data have been deposited in the Mass Spectrometry Interactive Virtual Environment (MassIVE) repository under accession MSV000100864 and are publicly available.

## Author contributions

ZS and YZ designed the study. ZS conducted experiments and acquired the data for most of the study. YZ, RJT, ENM, AD, IV, TT, EE, YS, YG, DPF, EJY, JH, ELL, JRH, JAS, KDB, and GJR assisted with experiments and data acquisition. RMK and PM provided resources and assistance. YZ and RDS provided resources and supervised the project. YZ conceived and wrote the manuscript. All authors read and approved the final manuscript.

## Conflict of interest

ZS, RJT, RDS, and YZ have a patent in filing related to the GPR182 research project titled, Methods of treating obesity related risorders by modulating GPR182 (US application no. 63/753267; PCT application no. PCT/US26/13504). YZ consults for DynamiCure Biotechnology.

## Funding support

This work is the result of NIH funding, in whole or in part, and is subject to the NIH Public Access Policy. Through acceptance of this federal funding, the NIH has been given a right to make the work publicly available in PubMed Central.

- National Cancer Institute (NCI), NIH R01 grants (CA269644, CA258302, and CA279398, to YZ).
- Mid-Career Bridge Grant from the Melanoma Research Foundation (to YZ).

## Supplementary Material

Supplemental data

Unedited blot and gel images

Supporting data values

## Version 1 03/24/2026

In-Press Preview

## Version 2 06/15/2026

Electronic publication
