# Supplementary material for: GPR182 is a lipoprotein receptor for dietary fat absorption
Source: J Clin Invest. 2026 Mar 24;136(12):e200857. doi: 10.1172/JCI200857 (PMC13262718; doi:10.1172/JCI200857)
Supplement: Supplemental data [file jci-136-200857-s171.pdf]

Supplementary Table 1 Serum cholesterol, triglycerides and free fatty acid in WT and GPR182$^{-/-}$ mice at different ages.

Gender	Females						Males					
Age (month)	2		5		8		2		5		8	
Genotype	WT (n=10)	*Gpr182*$^{-/-}$ (n=9)	WT (n=10)	*Gpr182*$^{-/-}$ (n=8)	WT (n=10)	*Gpr182*$^{-/-}$ (n=9)	WT (n=10)	*Gpr182*$^{-/-}$ (n=10)	WT (n=10)	*Gpr182*$^{-/-}$ (n=10)	WT (n=8)	*Gpr182*$^{-/-}$ (n=8)
Total Cholesterol (mg/dL)	83.22±7.49	97.85±9.08**	85.28±7.29	109.45±7.02****	93.63±10.15	111.72±7.22***	104.53±5.47	121.15±10.30***	110.17±9.63	137.91±17.41***	115.38±8.53	138.64±7.22****
Cholesteryl Esters (mg/dL)	49.93±5.04	64.98±6.18****	51.04±4.11	76.55±4.77****	56.41±7.48	77.98±6.28****	60.39±3.02	83.09±9.66****	63.48±7.15	97.64±10.96****	68.29±5.86	97.71±6.94****
Free Cholesterol (mg/dL)	33.29±3.13	32.87±3.23^{ns}	34.24±3.87	32.90±5.04^{ns}	37.22±4.19	33.74±3.63^{ns}	44.14±2.59	38.06±3.73***	46.69±5.68	40.27±7.04*	47.09±5.79	40.93±2.94*
Triglycerides (mg/dL)	82.14±5.19	75.66±6.37*	92.39±6.08	76.95±5.88***	104.21±9.37	81.54±7.27****	91.87±6.45	79.82±5.07***	102.85±8.30	84.47±6.53****	119.33±7.78	88.15±6.11****
Free Fatty Acid (mM)	0.67±0.05	0.61±0.04*	0.81±0.07	0.65±0.05****	0.91±0.08	0.71±0.07****	0.73±0.05	0.69±0.04*	0.99±0.08	0.72±0.06****	1.18±0.07	0.75±0.05****

Figure S1

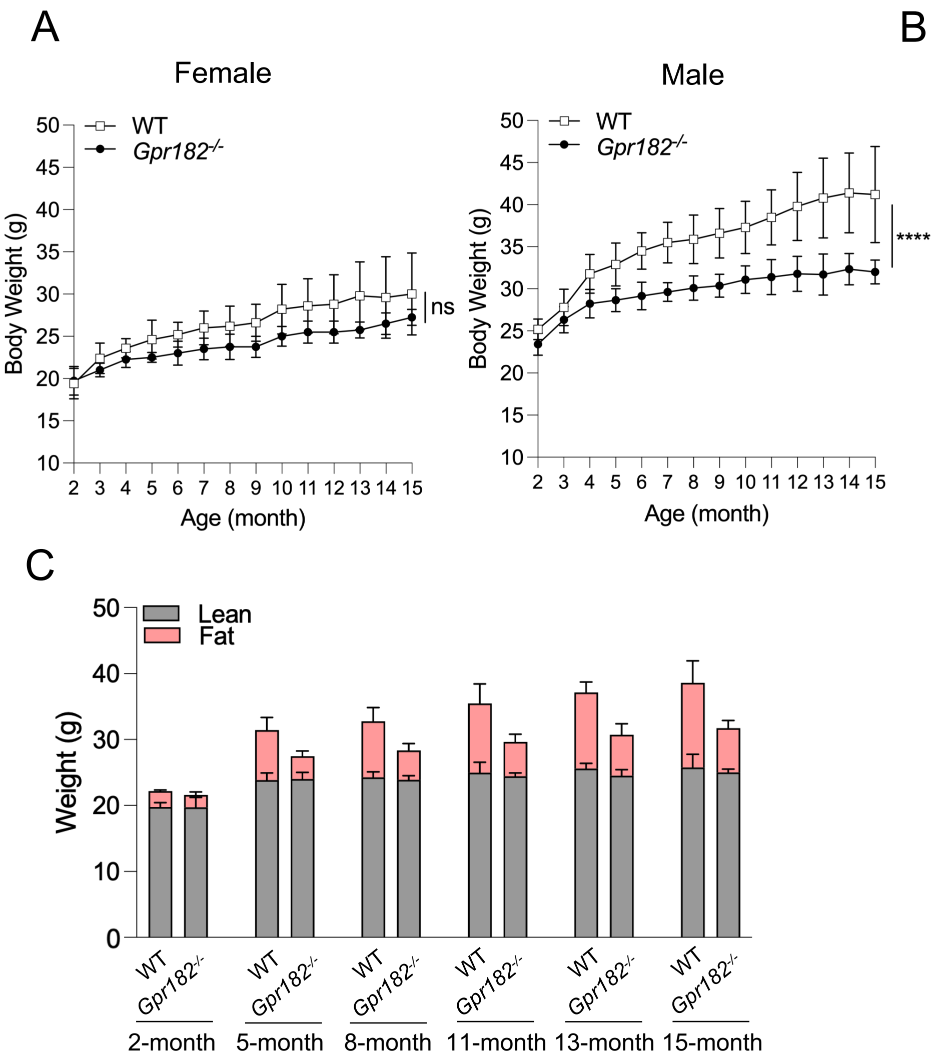

Supplementary Figure 1 *Gpr182$^{-/-}$* mice on regular chew diet are slimmer.

(A) 2-month-old WT and *Gpr182$^{-/-}$* mice on regular chow diet were weighed monthly over 13 months. Female, n=4, 5; Male, n=9, 10. (B) Representative images of male WT and *Gpr182$^{-/-}$* mice on chow diet at different ages. Scale bars: 1 cm. (C) Fat and lean masses of male WT and GPR182$^{-/-}$ mice on regular chow diet were assessed by MRI. n=5.

Figure S2

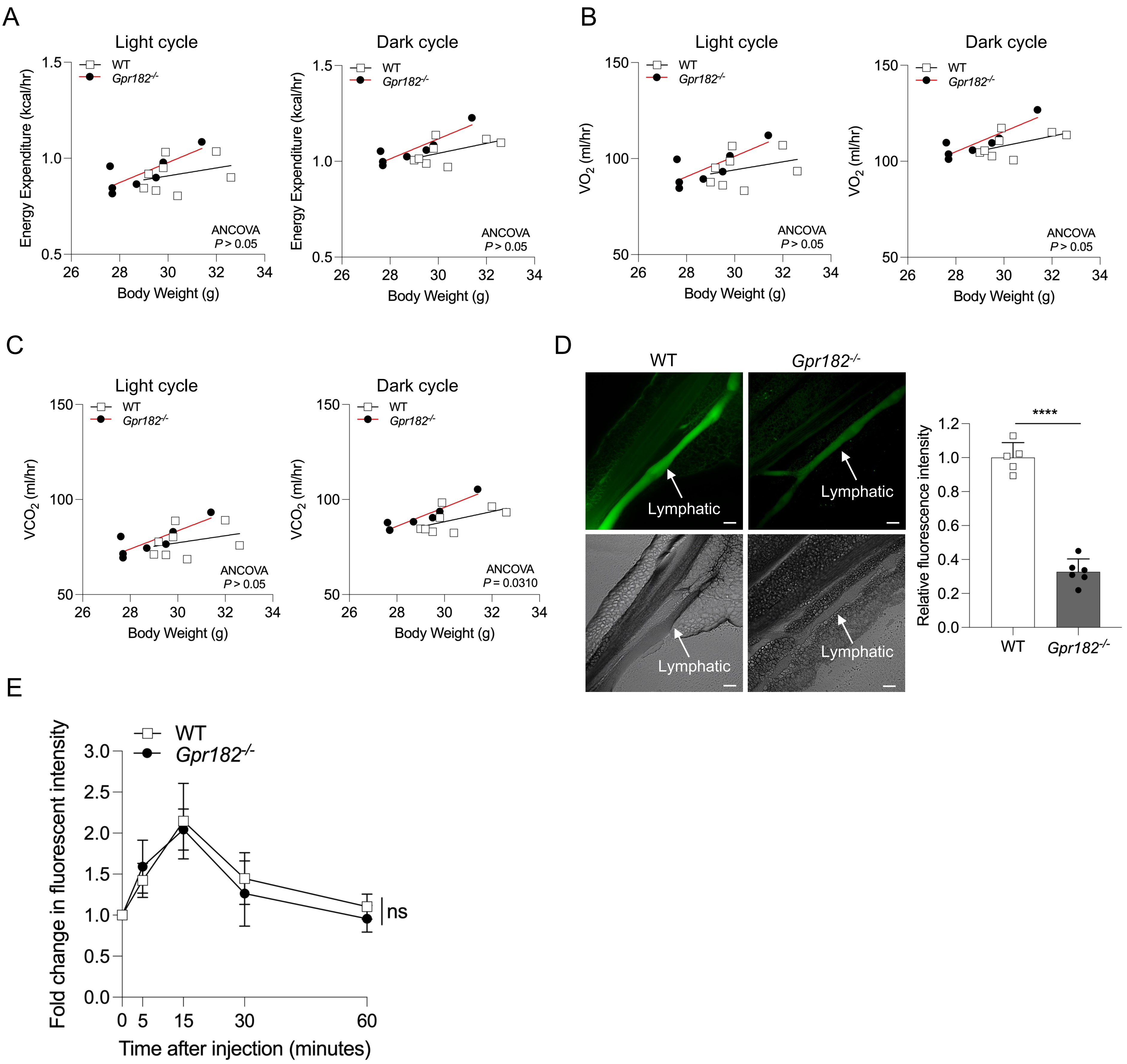

Supplementary Figure 2 *Gpr182*$^{-/-}$ mice exhibit selective defect in lipid absorption. (A-C) Male WT and *Gpr182*$^{-/-}$ mice at 2 to 3-month-old fed with HFD were assessed for energy balance in two weeks. n=7, 8. Energy expenditure (A), O_2 consumption (B) and CO_2 production (C) were plotted against body weight during the light cycle and dark cycle. (D) Representative fluorescent images of mesenteric lymph (white arrow) 2 hours after adult WT and *Gpr182*$^{-/-}$ mice were orally administrated with BODIFY-labeled C16 fatty acid. Fluorescence intensity in mesenteric lymph was quantified. n=5, 6. Scale bars: 100µm. (E) Adult female WT and *Gpr182*$^{-/-}$ mice under regular chow diet were injected with FITC-Dextran via Peyer's patches to assess draining function of intestinal lymphatics. Right after Dextran injection, blood samples were collected at different timepoints to measure fluorescence. n=5.

Figure S3

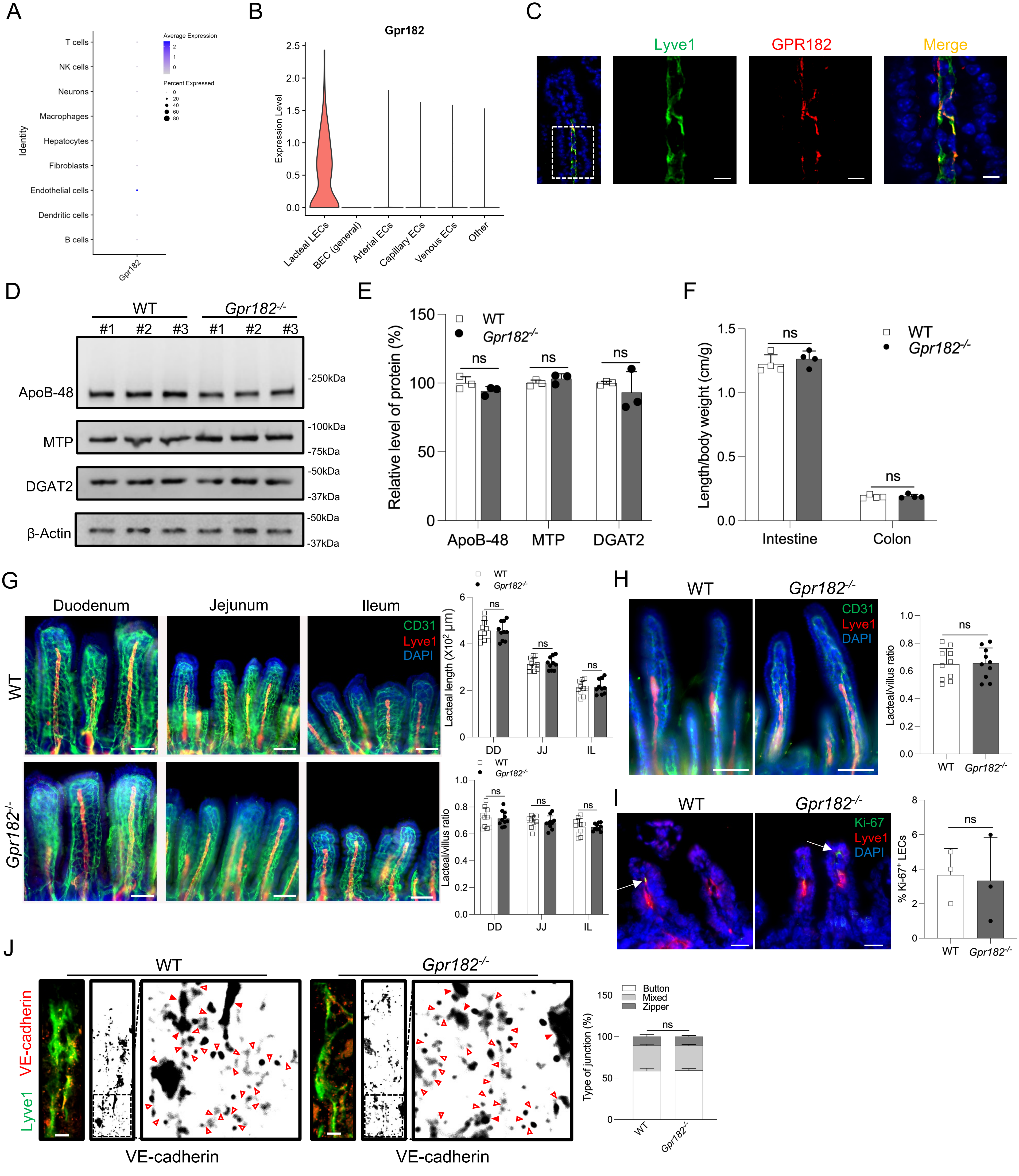

A
B
Gpr182
C
Lyve1
GPR182
Merge
D
WT
Gpr182-/-
ApoB-48
MTP
DGAT2
β-Actin
E
Relative level of protein (%)
F
Length/body weight (cm/g)
Intestine
Colon
G
Duodenum
Jejunum
Ileum
CD31
Lyve1
DAPI
Lacteal length (X10^2 μm)
Lacteal/villus ratio
H
I
Ki-67
% Ki-67+ LECs
J
VE-cadherin
Button
Mixed
Zipper
Type of junction (%)
ns

Supplementary Figure 3 Genetic knockout of GPR182 does not affect CM synthesis and lacteal structure. (A, B) Analysis of a public single-cell RNA sequencing dataset (PMID: 39567686) reveals that GPR182 expression in mouse small intestine is limited to a small population of ECs (A); additional analysis of EC population indicates that GPR182 expression is restrictedly found in lacteal LECs (B). (C) Immunofluorescent staining was performed to examine the expressions of GPR182 and Lyve1 in the small intestine. Scale bars: 10μm. (D, E) Western blot of female WT and *Gpr182*$^{-/-}$ small intestine was performed to examine the expressions of ApoB-48, MTP, and DGAT2. n=3. (F) The lengths of small intestine and colon in relate to body weight between adult WT and *Gpr182*$^{-/-}$ mice were compared. n=4. (G, H) Small intestines were stained with Lyve1 and CD31 to compare absolute and relative lacteal lengths between adult female (G) and P6 (H) WT and GPR182$^{-/-}$ mice. n=10. Scale bars: 100μm. (I) Representative images of Ki67 and Lyve1 staining in the small intestine of P6 newborns. Ki67^{+} LECs (arrows) were quantified. n=3. Scale bars: 20μm. (J) Representative images of VE-cadherin^{+} LEC junctions of Lyve1^{+} lacteals in the villi of adult male WT and *Gpr182*$^{-/-}$ mice. Zipper-like (red arrowheads) and button-like (open arrowheads) junctions were quantified. n=3. Scale bars: 40μm.

Figure S4

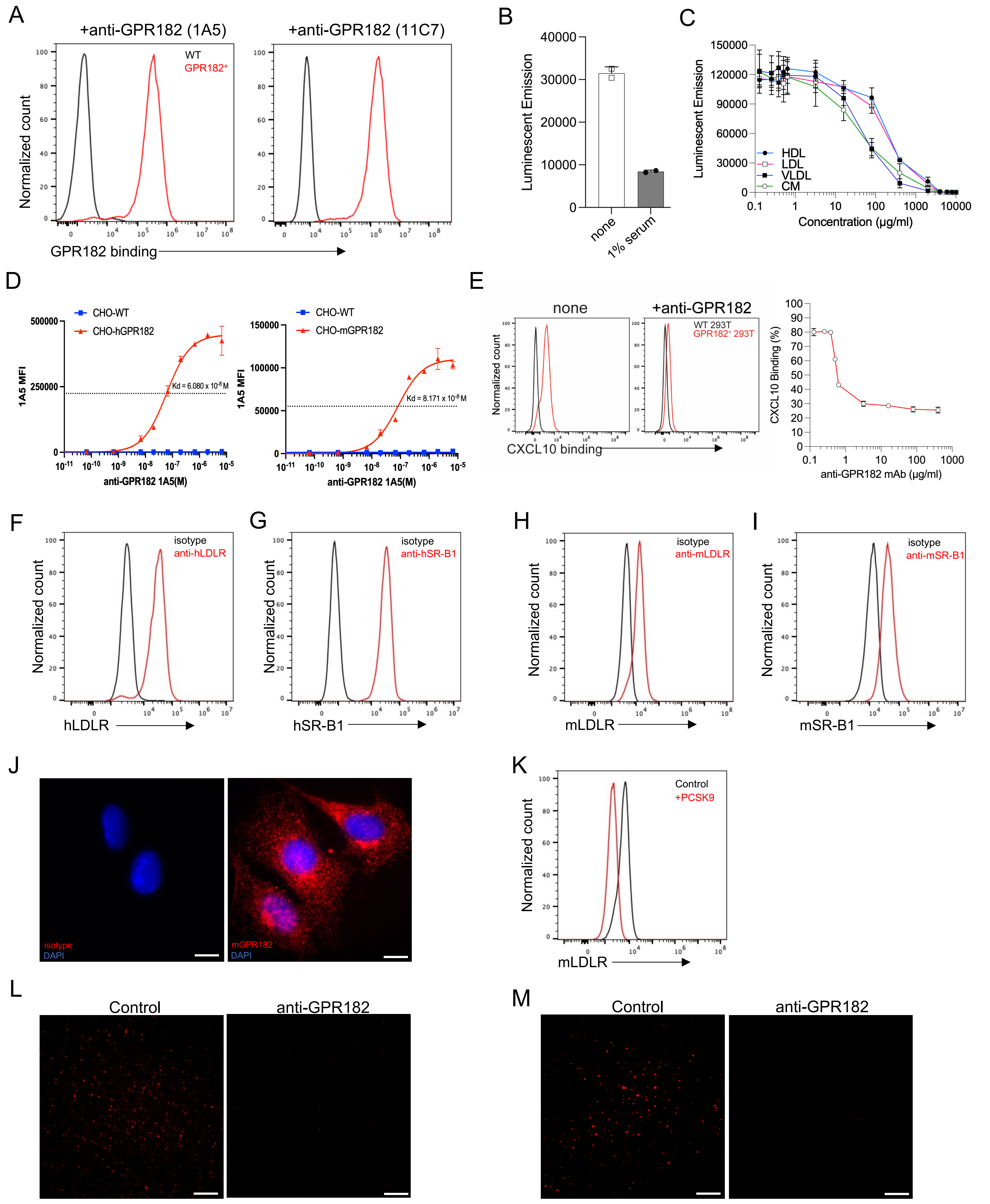
A
+anti-GPR182 (1A5)
+anti-GPR182 (11C7)
WT
GPR182⁺
Normalized count
GPR182 binding
B
Luminescent Emission
none
1% serum
C
Luminescent Emission
HDL
LDL
VLDL
CM
Concentration (μg/ml)
D
CHO-WT
CHO-hGPR182
CHO-mGPR182
1A5 MFI
Kd = 6.080 x 10-8 M
Kd = 8.171 x 10-8 M
anti-GPR182 1A5(M)
E
none
+anti-GPR182
WT 293T
GPR182⁺ 293T
Normalized count
CXCL10 binding
CXCL10 Binding (%)
anti-GPR182 mAb (μg/ml)
F
isotype
anti-hLDLR
hLDLR
G
isotype
anti-hSR-B1
hSR-B1
H
isotype
anti-mLDLR
mLDLR
I
isotype
anti-mSR-B1
mSR-B1
J
isotype
DAPI
mGPR182
DAPI
K
Control
+PCSK9
mLDLR
L
Control
anti-GPR182
M
Control
anti-GPR182

Supplementary Figure 4 Lipoproteins interact with GPR182. (A) The specificity of human GPR182 mAb 1A5 and 11C7 was verified by their specific binding to GPR182-expressing HEK293T cells. (B) In a NanoBit assay, human serum was added to evaluate its capacity of disrupting the GPR182/β2-arrestin association. (C) Similarly, serum lipoproteins were added at different concentrations to assess their effect in inhibiting the GPR182/β2-arrestin association. (D) Clone 1A5 binds to both human and mouse GPR182 strongly. CHO-hGPR182 (right) and CHO-mGPR182 cells were stained with titrated 1A5 by flow cytometry to determine the binding affinity. (E) Titrated 1A5 was tested for its ability to block the binding of $GPR182^{+}$ HEK293T cells by CXCL10-AF647. (F, G) HEK293T cells were assessed for the expressions of LDLR (H) and SR-B1 (I). (H, I) SVEC4-10 cells were assessed for the expressions of LDLR (H) and SR-B1 (I). (J) Immunofluorescent staining was performed to examine the expressions of mGPR182 in SVEC4-10 cells. (K) Upon treated with PCSK9, SVEC4-10 cells were assessed for the expression of LDLR. (L, M) Representative fluorescence images of Dil-HDL (L) and Dil-CM (M) in the lower chamber. Quantification was performed using a fluorescence plate reader (Fig. 4H); images were shown for visual verification only. Scale bars: 10μm.

Figure S5

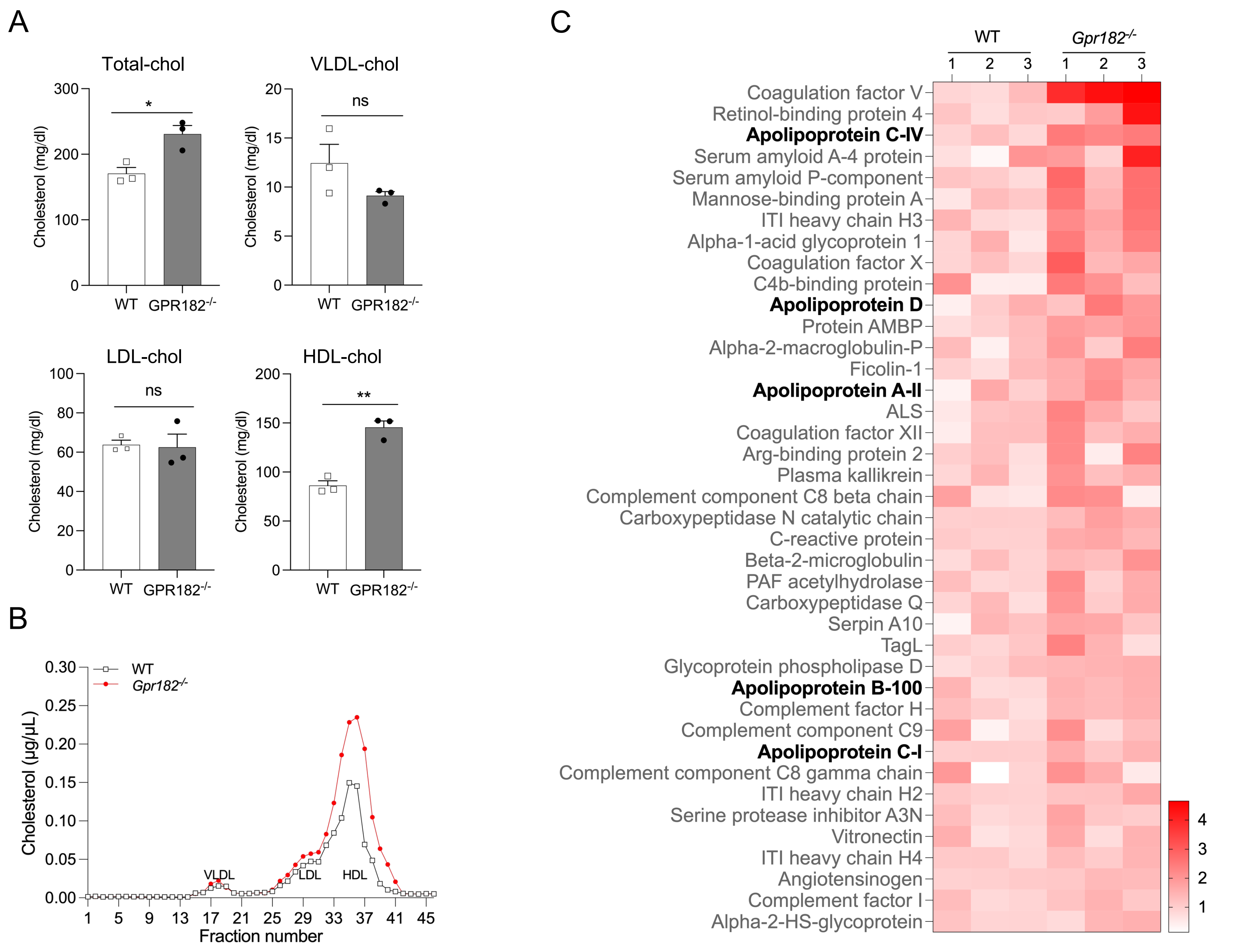

Supplementary Figure 5 GPR182 regulates lipoprotein homeostasis. (A) Serum lipoproteins in adult female *Gpr182*$^{-/-}$ and control WT mice were quantified by FPLC. n=3. (B) Pooled serum from four female WT and *Gpr182*$^{-/-}$ mice was applied to FPLC fractionation. Total cholesterol concentration was measured in each fraction. Fractions 15 to 20 were determined as VLDL, 25 to 30 as LDL, 31 to 41 as HDL. (C) Serum proteins in adult female WT and *Gpr182*$^{-/-}$ mice were determined by mass spectrometry. The top 40 serum proteins increased in *Gpr182*$^{-/-}$ mice after exclusion of immunoglobulins are shown, including several apolipoproteins. n=3.

Figure S6

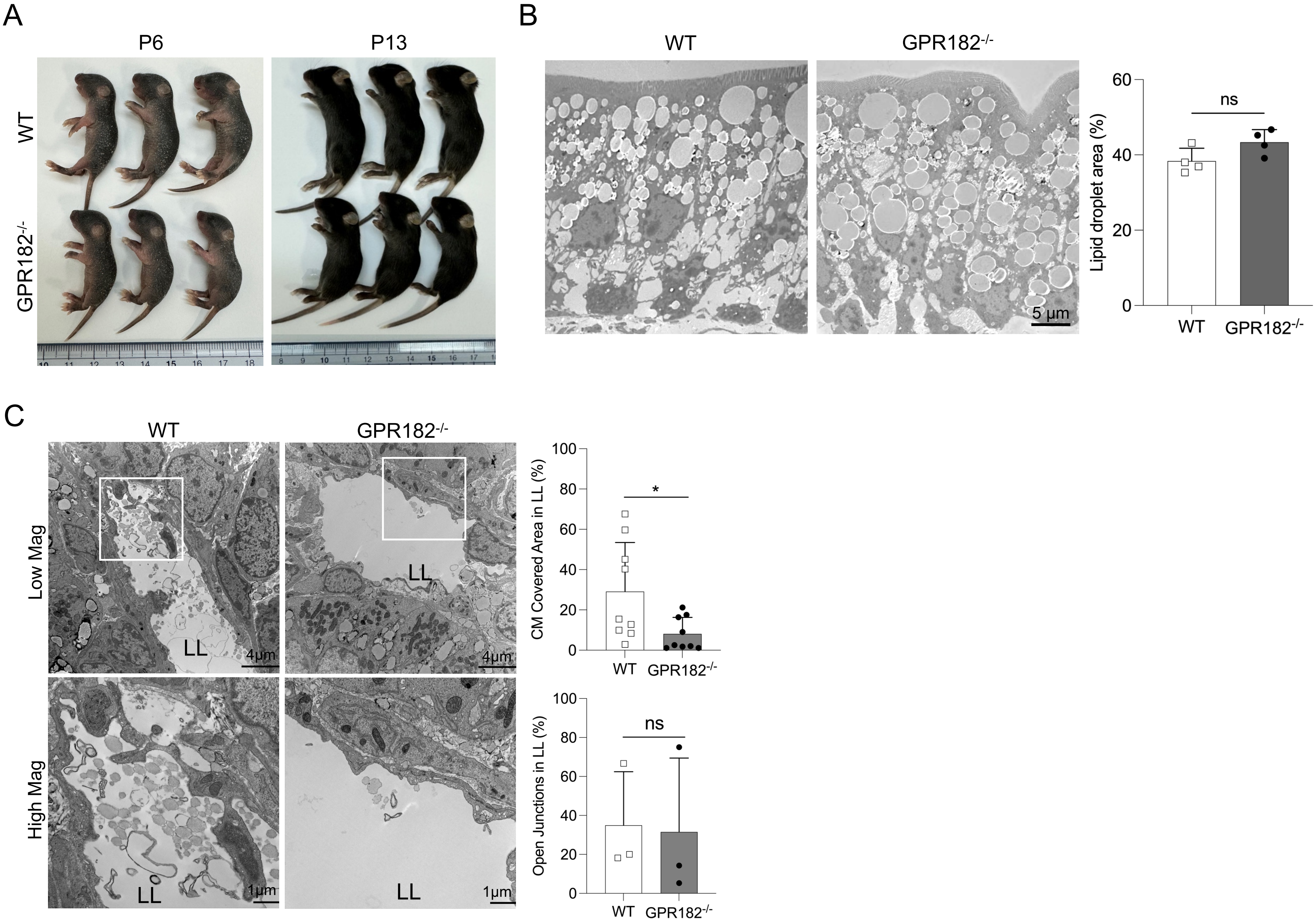

Supplementary Figure 6 GPR182 on LECs mediates fat absorption in the small intestine. (A) Images of WT and *Gpr182*$^{-/-}$ newborns (P6, P13) were recorded. (B) Representative TEM images of intestinal enterocytes 2 hours after oral oil gavage in female adult WT and *Gpr182*$^{-/-}$ mice. Lipid droplet area was quantified. n=4. Scale bars: 5μm. (C) TEM images of intestinal villus from P6 newborns of WT and *Gpr182*$^{-/-}$ mice. LL: lacteal lumen. CMs in lacteal lumens and open lacteal junctions were quantified. n=3. Scale bars: 4μm.

Figure S7

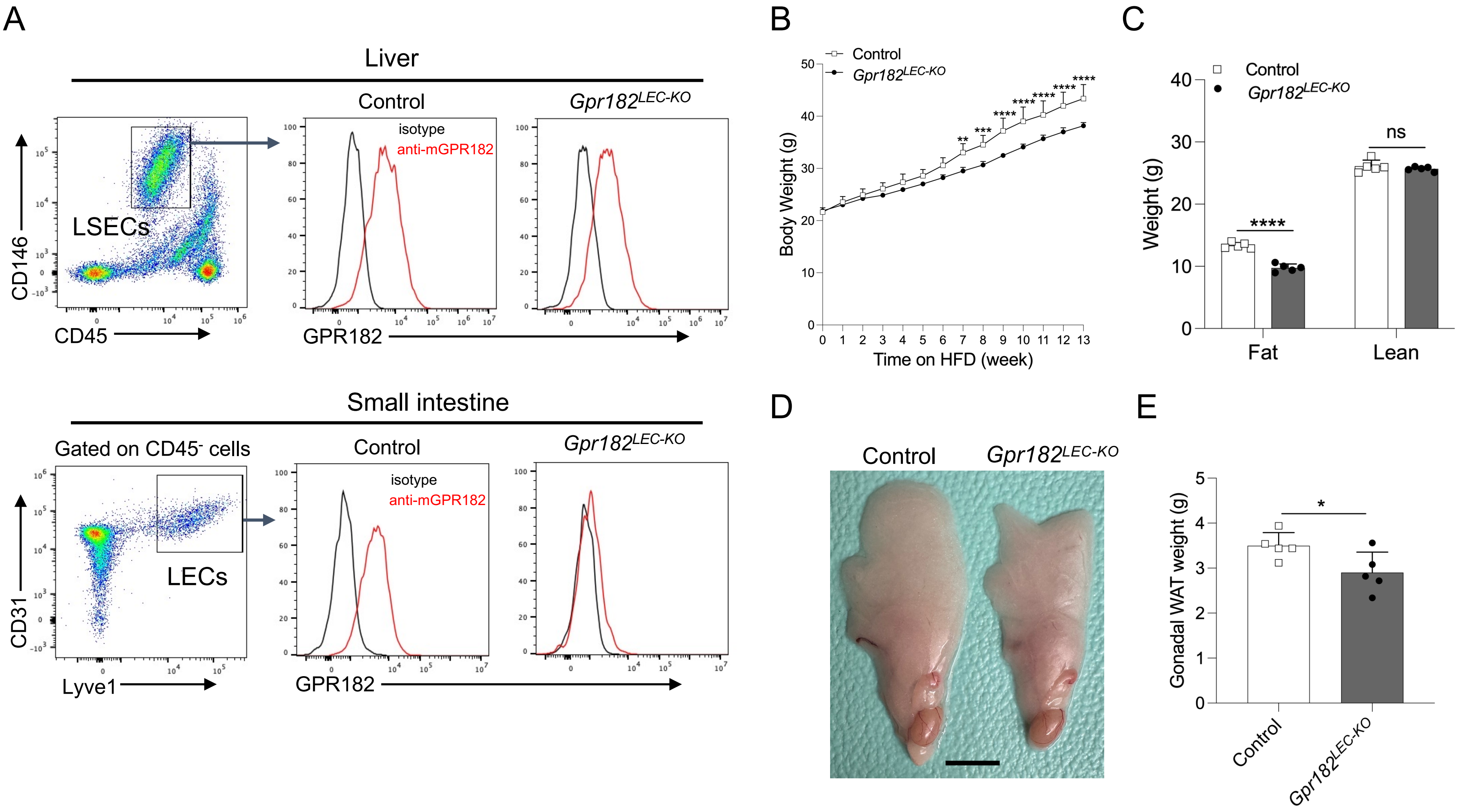

Supplementary Figure 7 LEC-specific *Gpr182*$^{-/-}$ mice are resistant to diet-induced obesity. 6-week-old male control *Gpr182*$^{fl/fl}$ and *Gpr182*$^{LEC-KO}$ mice were fed on HFD for 13 weeks before analysis. n=5. (A) Flow cytometric analysis of GPR182 expression in liver LSECs (CD45$^-$CD146$^+$) and intestinal LECs (CD45$^-$CD31$^+$Lyve1$^+$) from control and *Gpr182*$^{LEC-KO}$ mice. Representative histograms are shown. (B) Body weight was followed weekly. (C) Fat and lean masses of mice were determined by MRI. (D) Representative images of gonadal WATs were shown. (E) Gonadal WAT weight was determined. Scale bars: 1 cm.

Figure S8

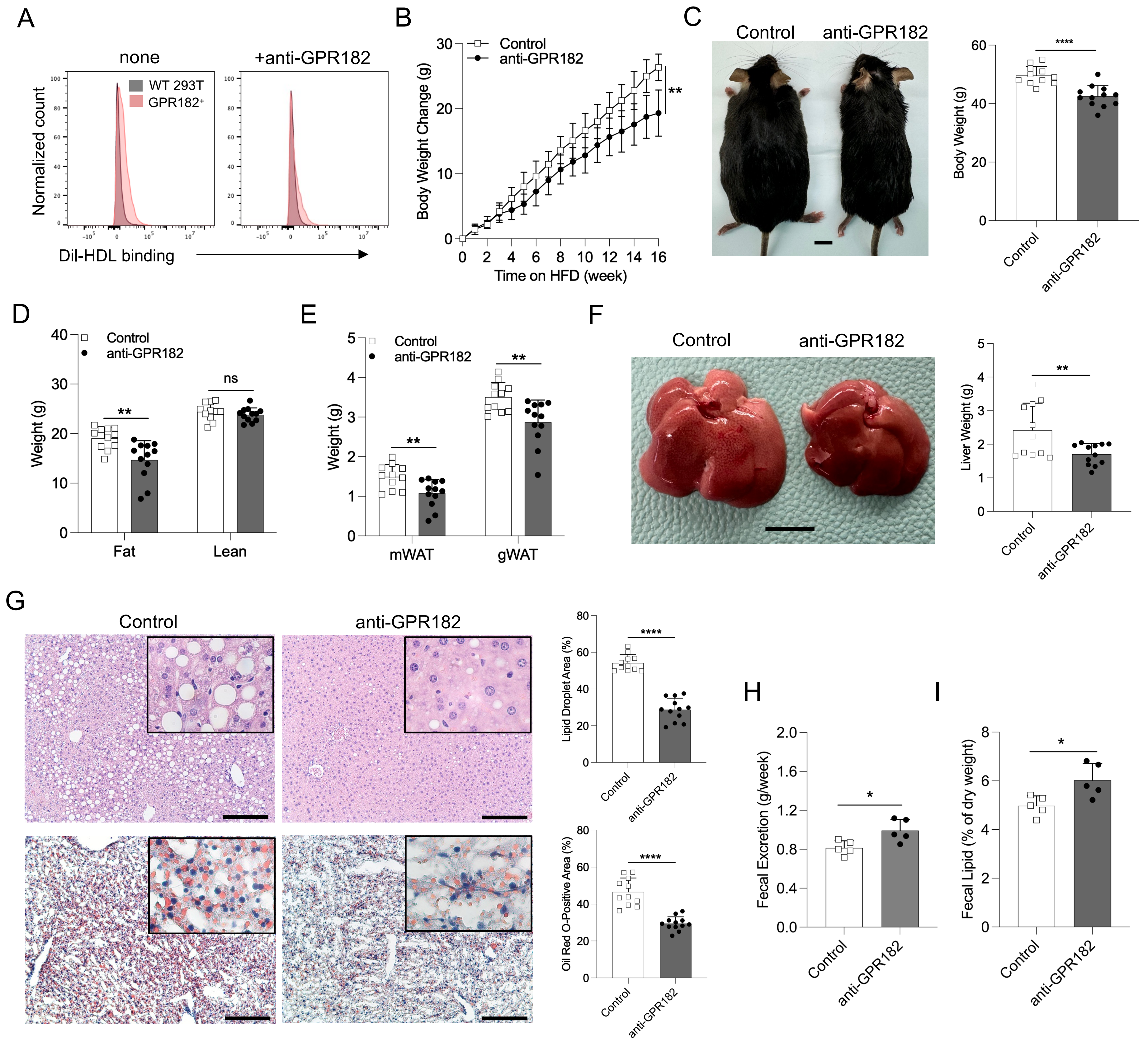

Supplementary Figure 8 Anti-GPR182 (clone 11C7) slows down HFD-induced obesity. (A) Human GPR182 mAb (clone 11C7) was assessed for its capacity of blocking Dil-HDL binding. (B-I) 7-week-old male hGPR182-KI mice were fed on HFD for 16 weeks. Mice were treated with control or anti-hGPR182 mAb weekly at the beginning of HFD feeding. n=11,12. (B) Body weight gain was followed weekly. (C) Representative images of hGPR182-KI mice upon 16 weeks of HFD feeding were shown and body weight was recorded. Scale bar: 1 cm. (D) Fat and lean masses of mice were determined by MRI. (E) gWAT and mWAT weights were determined. (F) Representative images of livers from antibody-treated mice on HFD were shown. Liver weight was calculated in the right panel. Scale bar: 1 cm. (G) H&E and Oil Red O staining of liver tissues from antibody-treated mice was recorded and quantified. Scale bars: 200 μm. (H) Weekly fecal excretion and (I) fecal lipids from hGPR182-KI mice under HFD were measured. n=5.

Figure S9

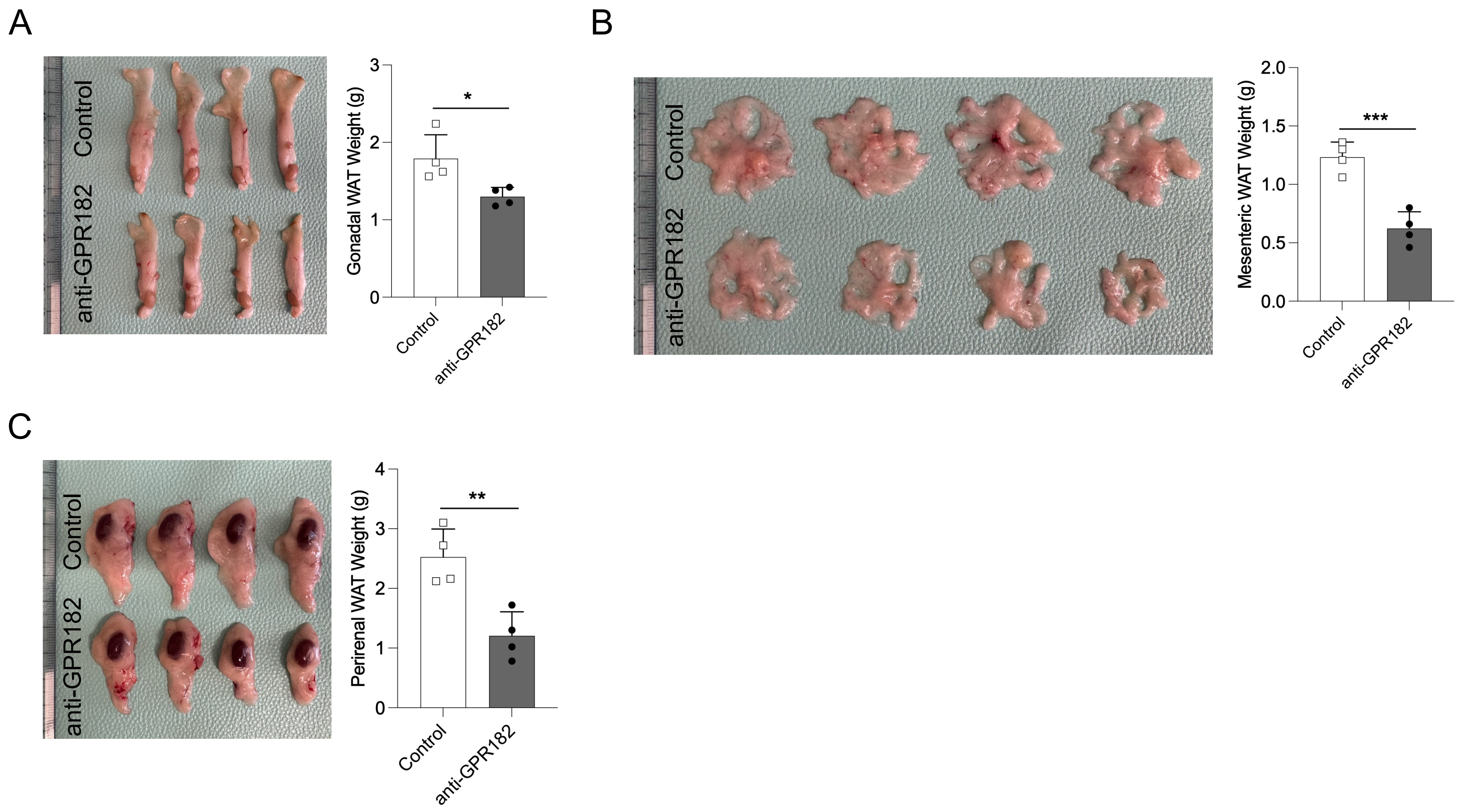

Supplementary Figure 9 Anti-GPR182 (clone 1A5) treats existing obesity. Male DIO WT B6 mice were treated with control or anti-GPR182 mAb (clone 1A5) for 8 weeks before analysis. (A) Images of gonadal WATs were shown. Gonadal WAT weight was determined in the right panel. (B) Images of mesenteric WATs were shown. Mesenteric WAT weight was determined in the right panel. (C) Images of perirenal WATs were shown. Perirenal WAT weight was determined in the right panel. n=4.
