# Supplementary material for: GPR182 is a lipoprotein receptor for dietary fat absorption
Source: J Clin Invest. 2026 Mar 24;136(12):e200857. doi: 10.1172/JCI200857 (PMC13262718; doi:10.1172/JCI200857)

Full unedited blot for Figure S3D

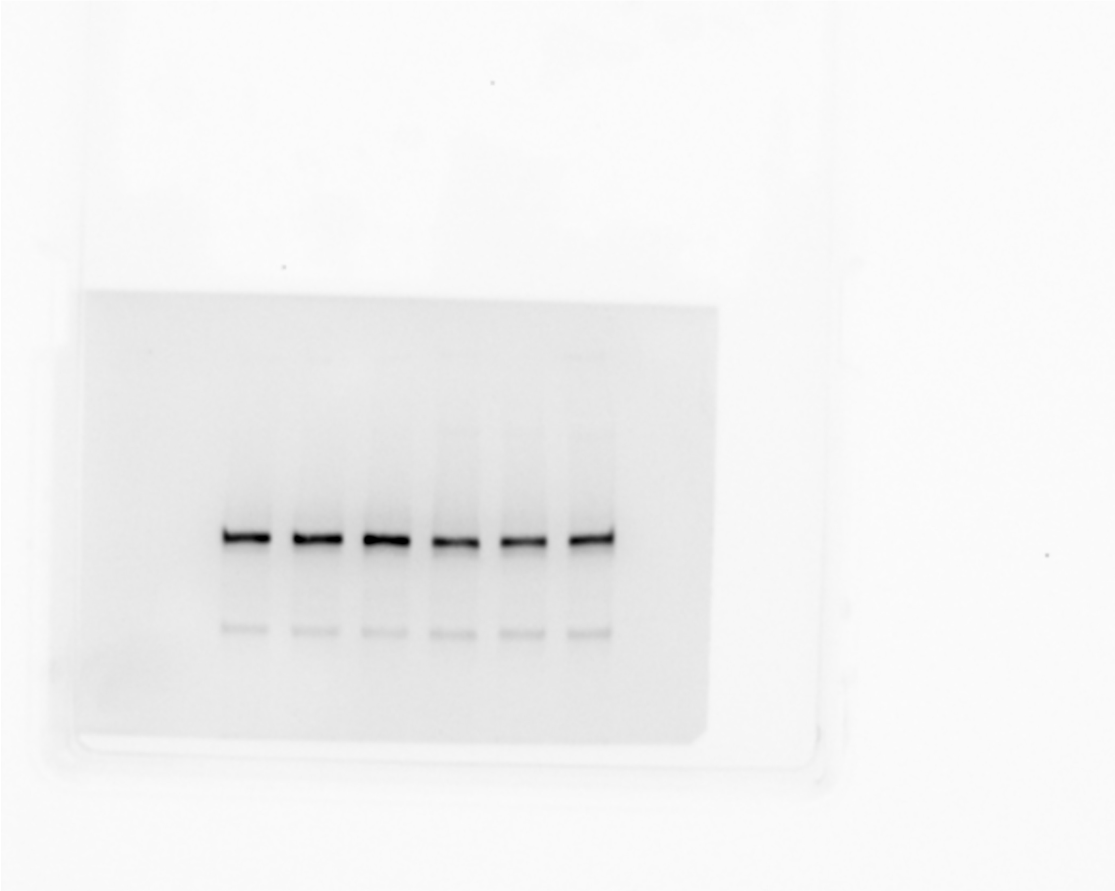

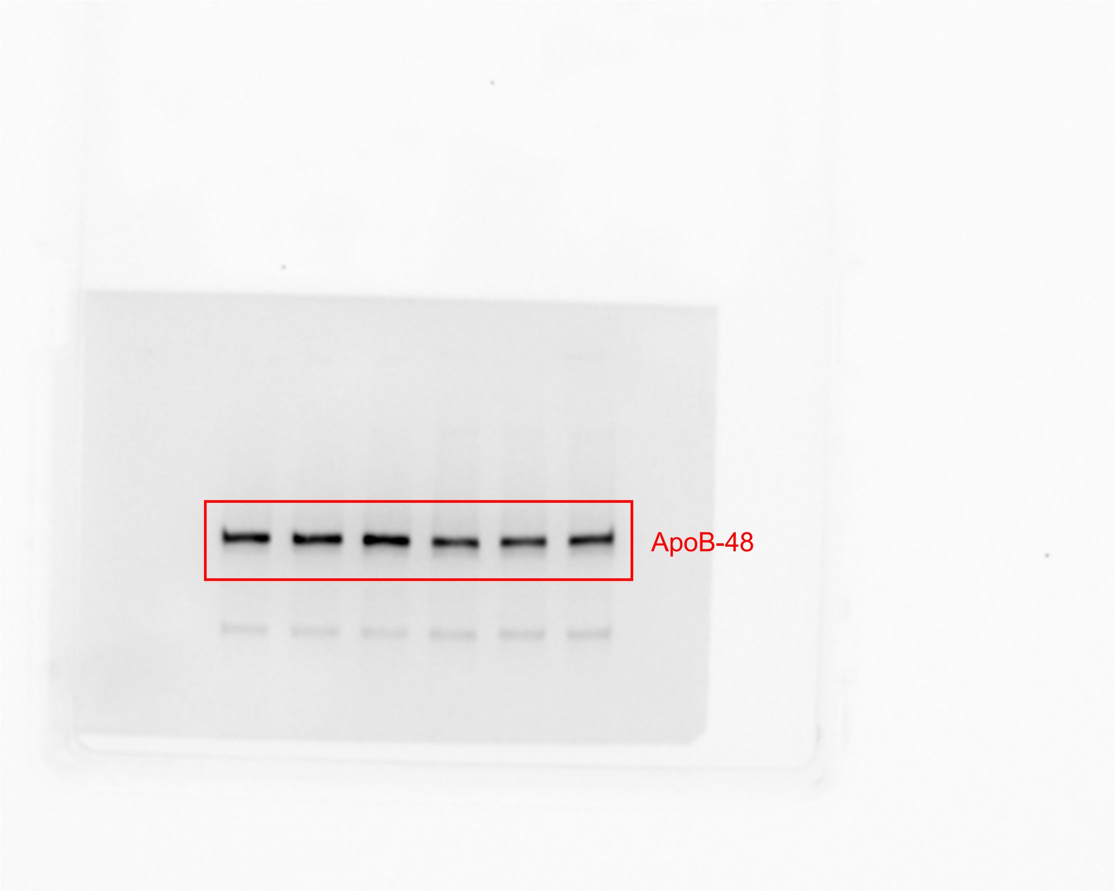

Full unedited blot for Figure S3D

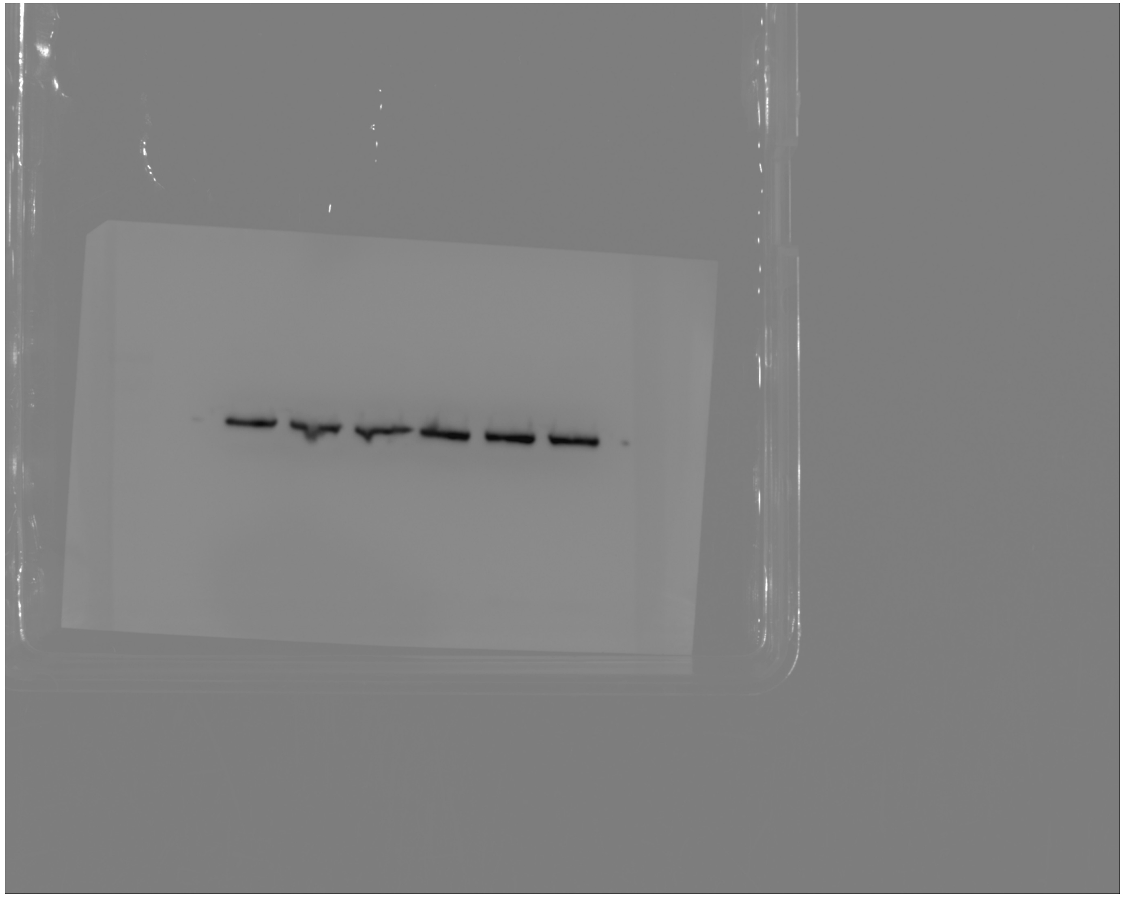

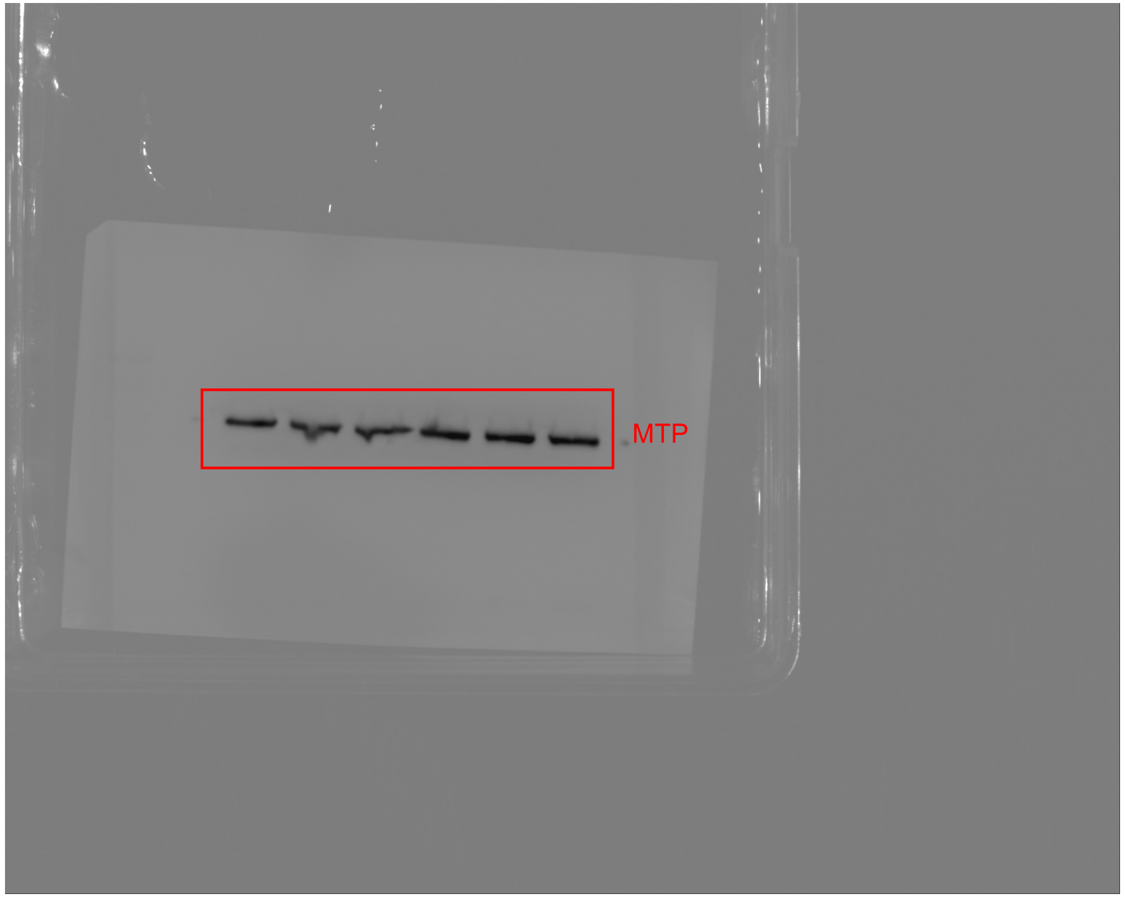

Full unedited blot for Figure S3D

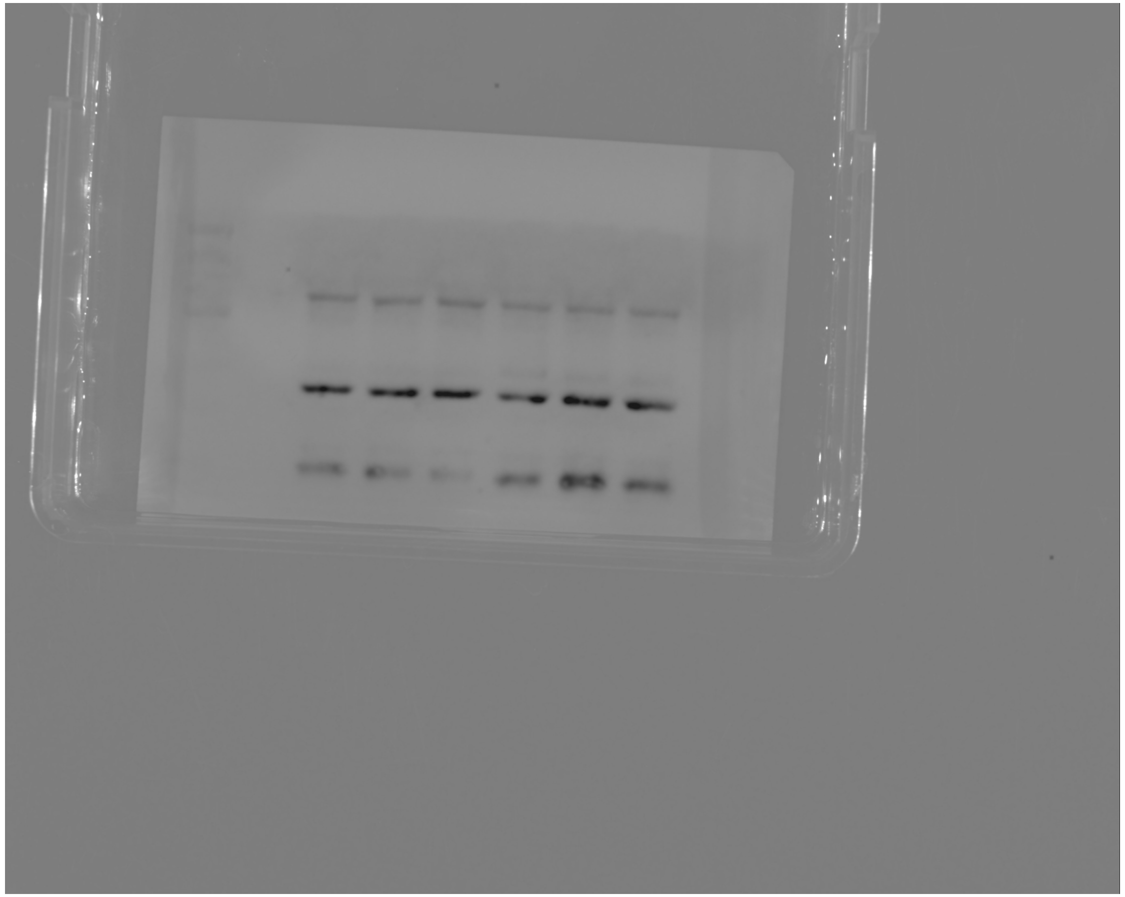

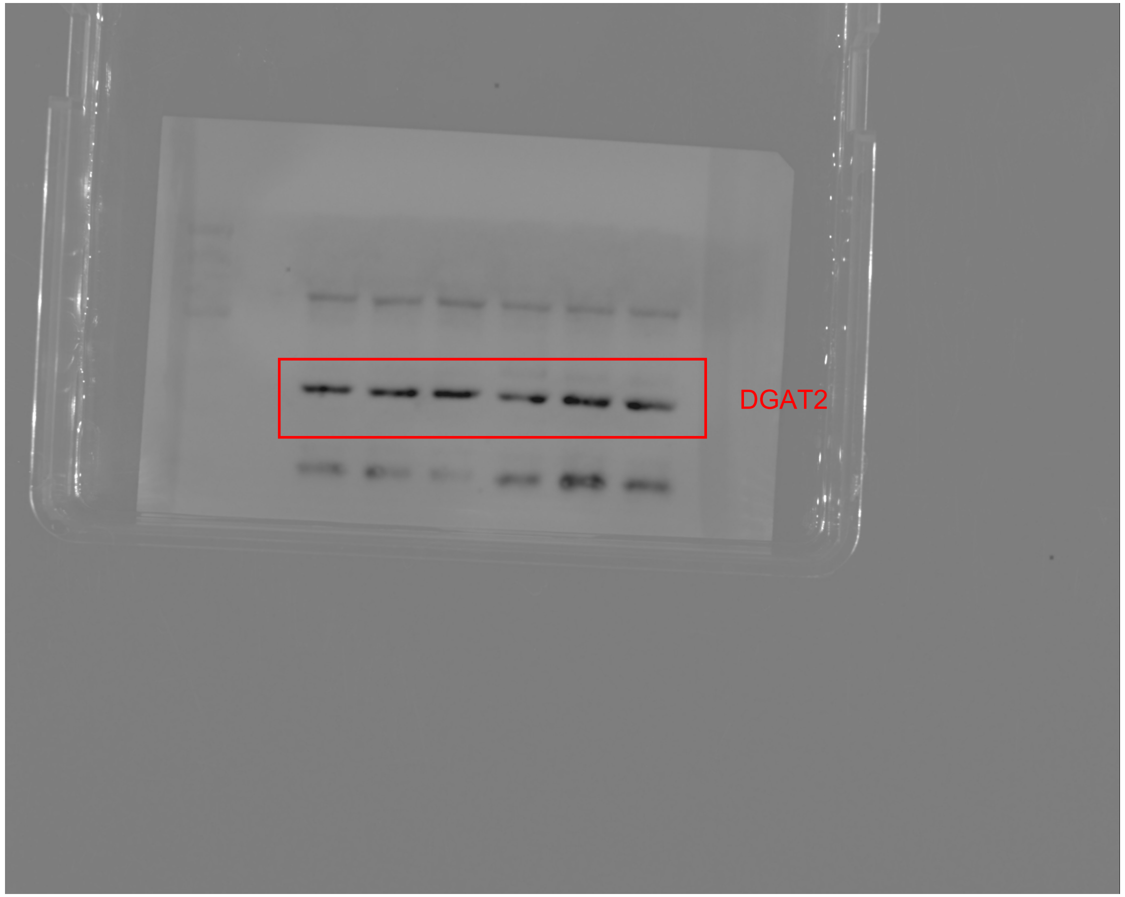

Full unedited blot for Figure S3D

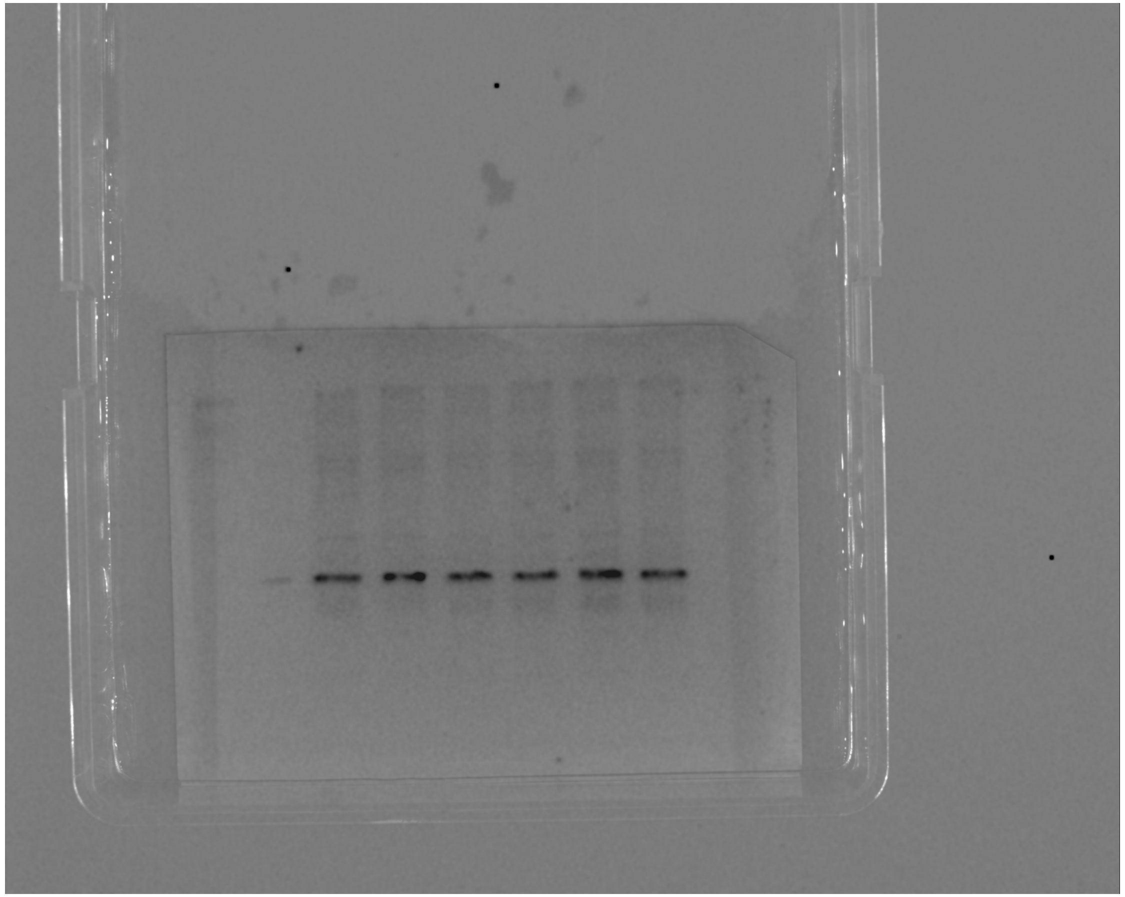

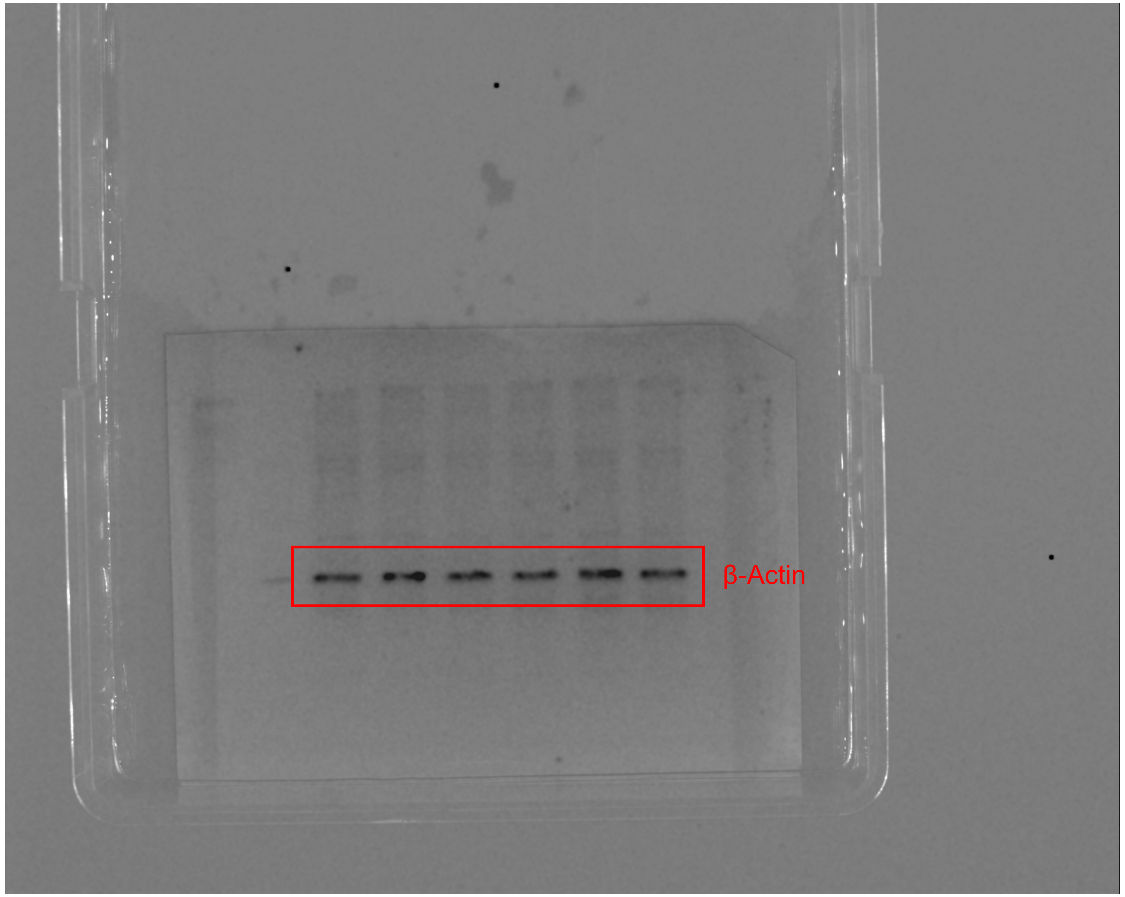

Supplement: Unedited blot and gel images [file jci-136-200857-s172.pdf]
